# Weak sharing of genetic association signals in three lung cancer subtypes: evidence at the SNP, gene, regulation, and pathway levels

**DOI:** 10.1186/s13073-018-0522-9

**Published:** 2018-02-27

**Authors:** Timothy D. O’Brien, Peilin Jia, Neil E. Caporaso, Maria Teresa Landi, Zhongming Zhao

**Affiliations:** 1 Vanderbilt Genetics Institute, Vanderbilt University School of Medicine, Nashville, TN USA; 2 Department of Biomedical Informatics, Vanderbilt University Medical Center, Nashville, TN USA; 3 Center for Precision Health, School of Biomedical Informatics, The University of Texas Health Science Center at Houston, 7000 Fannin St. Suite 820, Houston, TX 77030 USA; 4 Division of Cancer Epidemiology and Genetics, National Cancer Institute, Bethesda, MD USA; 5 Human Genetics Center, School of Public Health, The University of Texas Health Science Center at Houston, Houston, TX USA

**Keywords:** GWAS, eQTL, Enhancer, Lung cancer subtype, Functional genomics, Pathway analysis

## Abstract

**Background:**

There are two main types of lung cancer: small cell lung cancer (SCLC) and non-small cell lung cancer (NSCLC). NSCLC has many subtypes, but the two most common are lung adenocarcinoma (LUAD) and lung squamous cell carcinoma (LUSC). These subtypes are mainly classified by physiological and pathological characteristics, although there is increasing evidence of genetic and molecular differences as well. Although some work has been done at the somatic level to explore the genetic and biological differences among subtypes, little work has been done that interrogates these differences at the germline level to characterize the unique and shared susceptibility genes for each subtype.

**Methods:**

We used single-nucleotide polymorphisms (SNPs) from a genome-wide association study (GWAS) of European samples to interrogate the similarity of the subtypes at the SNP, gene, pathway, and regulatory levels. We expanded these genotyped SNPs to include all SNPs in linkage disequilibrium (LD) using data from the 1000 Genomes Project. We mapped these SNPs to several lung tissue expression quantitative trait loci (eQTL) and enhancer datasets to identify regulatory SNPs and their target genes. We used these genes to perform a biological pathway analysis for each subtype.

**Results:**

We identified 8295, 8734, and 8361 SNPs with moderate association signals for LUAD, LUSC, and SCLC, respectively. Those SNPs had *p* < 1 × 10^− 3^ in the original GWAS or were within LD (*r*^2^ > 0.8, Europeans) to the genotyped SNPs. We identified 215, 320, and 172 disease-associated genes for LUAD, LUSC, and SCLC, respectively. Only five genes (*CHRNA5*, *IDH3A*, *PSMA4, RP11-650 L12.2,* and *TBC1D2B*) overlapped all subtypes. Furthermore, we observed only two pathways from the Kyoto Encyclopedia of Genes and Genomes shared by all subtypes. At the regulatory level, only three eQTL target genes and two enhancer target genes overlapped between all subtypes.

**Conclusions:**

Our results suggest that the three lung cancer subtypes do not share much genetic signal at the SNP, gene, pathway, or regulatory level, which differs from the common subtype classification based upon histology. However, three (*CHRNA5, IDH3A,* and *PSMA4*) of the five genes shared between the subtypes are well-known lung cancer genes that may act as general lung cancer genes regardless of subtype.

**Electronic supplementary material:**

The online version of this article (10.1186/s13073-018-0522-9) contains supplementary material, which is available to authorized users.

## Background

Lung cancer is the second most commonly occurring cancer in the United States and is responsible for the most cancer-related deaths for both men and women, excluding data from skin cancer [1]. Although environmental risk factors such as smoking have major contributions to lung cancer development [2], there is also a genetic component, and heritability estimates of genetic risk for lung cancer range from 8% to 14% [3, 4]. Small cell lung cancer (SCLC) and non-small cell lung cancer (NSCLC) are the two main histological types of lung cancer [5]. The two main subtypes of NSCLC are lung adenocarcinoma (LUAD) and lung squamous cell carcinoma (LUSC). LUAD and LUSC comprise the vast majority of newly reported lung cancer cases, while SCLC comprises only a small subset (~15%) [6]. These subtypes differ in their location within the lung as well as the cell type of origin [7] and, therefore, may have different underlying disease etiologies. LUAD is the most researched lung cancer subtype, and studies have identified genomic alterations and actionable mutations [8–11]. Additionally, genomic alterations have been discovered in LUSC [12–14] and SCLC [15, 16]. Although it was discovered from these studies that few somatically mutated genes overlap all three subtypes, most of these studies focused on somatic mutations. Few studies have expanded the analysis to the germline genome.

In 2014, Hoadley et al. performed an integrative analysis to cluster cancers using 12 different cancer types from The Cancer Genome Atlas (TCGA) project [17]. They discovered that LUAD is a separate cluster and is distinct from LUSC, which clusters with the other squamous-like cancer types. In 2016, Campbell et al. compared somatic genomic alterations of LUAD and LUSC using over 1000 combined somatic tumor tissue samples [18]. They found that only six mutated genes overlapped both subtypes and that each subtype shared only about 25% of copy number alterations. Their work supports the conclusion that both subtypes are very distinct diseases. Common germline variation associated with lung cancer has also been studied for more than one subtype using genome-wide association studies (GWASs) [19–22].

Several GWASs have discovered common genetic variation associated with lung cancer risk [19–32]. However, few studies used data for all three subtypes [19, 20, 23, 26, 28, 30, 31]. Additionally, most of these findings did not reach the stringent genome-wide significance for a GWAS (*p* < 5 × 10^− 8^), and most of the genome-level significant single-nucleotide polymorphisms (SNPs) were located within non-coding regions of the genome, making it difficult to infer the underlying mechanism of the significant variants that could cause disease. Recent studies have shown that these marginally significant SNPs found from GWASs within non-coding regions of the genome may function in regulatory roles [33, 34]. Therefore, these results can be used to obtain a set of regulated genes to investigate and compare the similarity of the three lung cancer subtypes at the germline gene level and at the regulation level.

In this study, we first selected a set of SNPs with moderate association signals (*p* < 1 × 10^− 3^) from the summary results of a prior GWAS that covered three lung cancer subtypes (LUAD, LUSC, and SCLC). Then, we identified and compared regulatory variants associated with the three subtypes of lung cancer, as well as their target genes. We used these results to investigate the similarity of the subtypes at the SNP, gene, regulatory, and pathway levels. We first remapped these SNPs to an updated human genome reference (hg19) and expanded them using linkage disequilibrium (LD) patterns from a European population. We used this final set of SNPs to examine several lung tissue expression quantitative trait loci (eQTL) and enhancer datasets for evidence of a regulatory function for each SNP and identified their target genes. We compared the target genes of these regulatory SNPs and observed that only five genes overlapped all three subtypes. We also observed a weak overlap among all three subtypes across all comparisons. Through this analysis, we identified many genes that might have an important association with lung cancer for each specific subtype. Follow-up studies on these genes may lead to a better understanding not only of the genes themselves, but also the underlying biology that differentiates these subtypes of lung cancer. Our results provide insights into the distinct genetic components among the three lung cancer subtypes.

## Methods

### GWAS dataset

We previously performed a multi-site GWAS for lung cancer in a European population and analyzed each sample by lung cancer subtype for the National Cancer Institute’s GWAS for lung cancer (more details are available in the original publication [20]). Briefly, this GWAS for lung cancer used cases and controls from four different studies: Environment and Genetics in Lung Cancer Etiology (EAGLE), Alpha-Tocopherol, Beta-Carotene Cancer Prevention (ATBC), Prostate, Lung, Colon, Ovary (PLCO) screening trial, and Cancer Prevention Study II (CPS-II). After the quality control of the genotyping results, there remained 5739 cases and 5848 controls of European ancestry and 515,922 SNPs. The analysis was stratified by lung cancer subtype with 1730 LUAD cases, 1400 LUSC cases, 678 SCLC cases, and 5848 shared controls. It used unconditional logistic regression. We used the full set of significant lung cancer SNPs (*p* < 1 × 10^− 3^) separated by subtype for this analysis.

### Genomic annotation of SNPs

The online web tool SNP Nexus [35] (http://snp-nexus.org/) was used to annotate the genomic location of the significant SNPs by lung cancer subtype based on the NCBI36/hg18 genome assembly. We used the UCSC hg18 gene definitions for the genomic annotation of each region.

### Conversion of hg18 SNPs to hg19 SNPs

The results from the lung cancer GWAS were originally generated using coordinates from the hg18 reference of the human genome. We converted these SNPs to hg19 coordinates using the online tool Remap from the National Center for Biotechnology Information (NCBI) with default settings (http://www.ncbi.nlm.nih.gov/genome/tools/remap). This conversion allowed us to map the SNPs to the regulatory annotation information, which were based on hg19 coordinates.

We used these updated hg19 coordinates for the SNPs to obtain the updated SNP rsID numbers using dbSNP data (build 142) from NCBI to account for any SNPs that may have been merged between assemblies.

### Identification of SNPs in LD with the genotyped SNPs

For each SNP, we retrieved all other SNPs in a 1-Mb region both upstream and downstream from the SNP site using Tabix [36] (version 0.2.5). We obtained the SNP data from the European super population group from the 1000 Genomes Phase III data (v5.20120502). Vcftools [37] (version 0.1.12b) was used to convert the Tabix vcf files to the plink-tped file format. Then we used the 1000 Genomes data for each SNP and applied PLINK [38] (version 1.07) to identify the final set of SNPs that were in the same LD with the tagging SNPs using *r*^2^ > 0.8 with 1 Mb upstream and downstream of the SNP. The LD results from PLINK were combined for every SNP and any SNPs in LD that were duplicated across all SNP sets were removed.

### Randomization for overlapping SNPs

All LD-based pruning of SNPs was performed using the PLINK formatted 1000 Genomes Phase III European dataset. To identify a set of more independent SNPs (more independent and not purely independent) in each subtype, SNPs with *p* < 1 × 10^− 3^ from the GWAS summary results were extracted for LUAD, LUSC, and SCLC, and PLINK was used to prune out a set of SNPs with no strong linkage using the *indep-pairwise* function. The *r*^2^ used for all LD trimming was 0.5. These results were used to identify the new overlap of SNPs between LUAD, LUSC, and SCLC. To trim the background set of SNPs for the randomization, the entire set of SNPs genotyped and reported for each subtype was imported into PLINK with the same function and options.

The same number of SNPs for each subtype were randomly selected 10,000 times from the background pool of SNPs without strong linkage from the genotype chip in R. For each random selection, we determined the number of overlapping SNPs to identify the level of overlap that may occur by chance.

### Genotype-Tissue Expression eQTLs

The full set of significant human-tissue-specific eQTLs version 6 (V6) was downloaded from the Genotype-Tissue Expression (GTEx) website (https://www.gtexportal.org) on 22 February 2016. The eQTLs were identified using linear regression with the tool Matrix eQTL [39] with a ±1-Mb region around the transcription start site in each individual tissue that had >70 samples. The significance of the eQTLs was determined by empirical *p* values using permutations followed by a Storey false discovery rate. The eQTLs with a *q* value ≤5% were considered significant.

We also downloaded the full set of all multi-tissue eQTLs for nine different tissue types for the pilot phase of the GTEx Project on 11 June 2015. This file contained eQTLs discovered using two different methods, the University of Chicago model [40] and the University of North Carolina model [41], which are fully explained in the respective publications. Additionally, a file was included that contained the average between both methods including calculated posterior probabilities for every gene–SNP pair titled res_final_amean_com_genes_com_snps_all.txt. The whole SNP set (including LD SNPs) was used to detect eQTLs in this dataset. We plotted the distribution of posterior probabilities of all the eQTLs found using the SNPs and defined an eQTL as significant if its posterior probability was >80% (Additional file 1: Figure S1). We removed all duplicated genes in each subtype to obtain the final GTEx set of genes.

### Lung tissue eQTLs from the Hao et al. study

Hao et al. [42] investigated how genetic variation affects gene expression levels in human lung tissues. They used this dataset to interrogate SNPs associated with asthma. The authors used lung tissue and blood from more than 1000 patients across three cohorts to identify a set of eQTLs in lung tissue. We downloaded the entire set of cis-eQTLs identified from this study with the false discovery rate at 10%. We removed the target genes without annotated gene names. We also merged duplicate probes that specified the same genes into a single gene.

### FANTOM5 transcribed enhancers

The FANTOM consortium aims to identify and assign regulatory function to the mammalian genome. Part of this comprehensive project is to identify all transcribed enhancers and promoters in multiple human cell lines and tissue types. The entire set of permissive enhancers found in the FANTOM5 data was downloaded in bed file format from http://enhancer.binf.ku.dk/presets/permissive_enhancers.bed on 26 August 2015. The gene-report function was used in PLINK to search for any SNPs that were located within permissive enhancer regions. SNPs that were located within each enhancer region were then matched with the set of correlated expressed promoters for FANTOM5 enhancer transcription start sites downloaded from http://enhancer.binf.ku.dk/presets/enhancer_tss_associations.bed on 25 August 2015.

### IM-PET predicted enhancers

He et al. [43] developed a novel approach for identifying the target genes of histone-derived enhancers using a random forest classifier. The authors used this tool, Integrated Methods for Predicting Enhancer Targets (IM-PET), to define a set of enhancer target genes for 12 different cell types. We used the results for two lung cell types, IMR90 and NHLF, for our analysis. We used bedtools [44] version 2.17.0 to identify lung cancer SNPs within the enhancer regions that had an associated target gene. To remove non-expressed genes, we filtered the results to remove target genes with reads per kilobase per million mapped reads of 0. The enhancer targets were originally formatted as Ensembl-defined transcripts, so we converted them to gene symbols using the BioMart tool from Ensembl using the archived site pertaining to genome assembly GRCh37.p13 [45].

### Identification of independent loci for the identified germline genes

biomaRt [46] was used to annotate the genomic locations for the germline-regulated genes discovered from each dataset for each subtype using gene start and stop coordinates from Ensembl gene definitions using genome build GRCh37.3. Genomic locations that were not defined from Ensembl were manually annotated using NCBI’s Gene online web resource https://www.ncbi.nlm.nih.gov/gene. The function cluster from bedtools [44] was used to cluster the genes into independent 1-Mb regions.

### Pathway enrichment analysis

The final set of germline-regulated genes was uploaded to the WebGestalt online resource [47]. The hypergeometric test was used for enrichment with specific pathways followed by Benjamini and Hochberg multiple test correction [48].

### GWAS Catalog SNPs

We downloaded all SNPs from the GWAS Catalog using the search term “lung cancer” on 13 January 2016. We removed SNPs where the initial or replication population was other than European. We also removed SNPs that were reported in the original lung cancer report [20] because they were used in our analyses.

### Principal component analysis of TCGA germline genotyped SNPs

TCGA germline genotype level 2 data for six cancer types (LUAD, LUSC, head and neck squamous cell carcinoma, bladder urothelial carcinoma, glioblastoma multiforme, and lower grade glioma) were downloaded from the legacy archive of the data portal of the National Cancer Institute’s Genomic Data Commons using its data transfer tool after obtaining permission from the database of Genotypes and Phenotypes (dbGaP). The normal blood samples were extracted from these sets to use in the analyses. These normal blood samples were then filtered to exclude non-white and white Hispanic samples as defined by the clinical data from TCGA. These birdseed genotype formatted files were then altered for use in PLINK as follows. First, all low-confidence calls (confidence > 0.1) were initially recoded as −9 in place of the 0,1,2 allele birdseed conventions. Second, each sample was merged together for each cancer type to obtain a matrix of the number of genotyped SNPs (906,600) times the number of samples. Third, the hg19/b37 Affymetrix mapping file was downloaded from the Affymetrix website and merged with the probe IDs in the birdseed matrices. Fourth, the Affymetrix annotation file was used to generate a PLINK format map file. Fifth, the alleles in the birdseed files were recoded to their appropriate bases according to the Affymetrix annotation file. After converting the files to PLINK format, all samples across all cancer types were merged together into one matrix using PLINK’s merge-list function. Tri-allelic SNPs were further removed to obtain the final merged genotype matrix.

To run the principal component analysis (PCA) function in PLINK, the SNPs were first filtered by LD *r*^2^ 0.5 using 1000 Genomes Phase III European only data downloaded from the VEGAS2 website. After an LD trim, the PCA function was run on all samples. After visualization of the top two principal components, we determined a set of outliers from multiple cancer types and ran the PCA function again after removing these outliers.

### Analysis of the overlap of SNPs and gene sets

The R package UpSetR [49] was used to make the plots of the overlapping SNPs and gene sets.

## Results

### Description of data

We obtained SNPs (*p* < 1 × 10^− 3^) for three lung cancer subtypes, LUAD, LUSC, and SCLC, from the results of the National Cancer Institute’s GWAS for lung cancer [20]. This GWAS utilized cases and controls from four smaller studies: Environment and Genetics in Lung Cancer Etiology (EAGLE), Alpha-Tocopherol, Beta-Carotene Cancer Prevention (ATBC), Prostate, Lung, Colon, Ovary (PLCO) screening trial, and Cancer Prevention Study II (CPS-II) nutrition cohort. Table 1 shows the total number of cases genotyped for each subtype, the total number of SNPs discovered by selection criterion (*p* < 1 × 10^− 3^), and the distribution of their locations within the genome. We also show the overlap of SNPs per subtype in Additional file 1: Figure S2A. We found that, like many GWASs for various disease types, only 2–3% of variants were located within coding regions of the genome.

**Table 1:** Summary of data from lung cancer genome-wide association studies

Subtype	Sample size	SNPs (*p* < 1 × 10^− 3^)				
		Total SNPs	Coding	Intron	UTR	Intergenic
LUAD	1730	544	13	228	7	296
LUSC	1400	598	18	299	16	265
SCLC	678	558	14	247	10	287

*LUAD* lung adenocarcinoma, *LUSC* lung squamous cell carcinoma, *SCLC* small cell lung cancer, *SNP* single-nucleotide polymorphism, *UTR* untranslated region

### Weak sharing of genetic association signals

A direct comparison using genotyped SNPs revealed that ten SNPs at *p* < 1× 10^− 3^ overlapped among all three subtypes (Additional file 1: Figures S2A and S3). To determine if this overlap of SNPs was different from what would be expected by chance, we conducted a randomization test through random resampling of the genotyped SNPs on the GWAS chip, which did not require individual genotyping data (see “Methods”). To avoid a randomization analysis that could be biased due to SNPs in strong LD, we pruned the original 515,922 SNPs as genotyped on the chip (Illumina HumanHap 550) that passed a quality check to obtain a set of SNPs that are assumed unrelated or weakly related at *r*^2^ = 0.5 (see “Methods”). These SNPs (234,859 after LD pruning) served as the pool for our randomization test. We provide the details of this SNP selection in Additional file 1: Figure S3. After LD pruning of these SNPs, we discovered that only one SNP, compared to ten SNPs from the original list, overlapped across all three lung cancer subtypes (Additional file 1: Figure S2C). This one SNP was rs578776, on chromosome 15 in the 3' untranslated region of *CHRNA3,* in the chr.15q25 locus known to be associated with different histology subtypes of lung cancer [50]. For the randomization test, we randomly chose the same number of SNPs for each subtype after LD pruning 10,000 times. Each time, we compared the three randomly selected size-matched sets of SNPs representing three subtypes and recorded the overlapping SNPs. After the 10,000 randomization trials, we observed ten times that one SNP overlapped among the three random sets of SNPs (10/10,000), while in the remaining 9990 sets, no overlap was observed (Additional file 1: Table S1). We observed no instances of an overlap greater than one SNP. Given the large number of SNPs in the pool, it was expected that there would not be many overlapping SNPs. Thus, the discovery of one overlapping SNP among the three lung cancer subtypes is likely within random expectation with a chance of 0.001 according to our randomization test. Therefore, we conclude that there is no strong evidence that the one overlapping SNP we observed is higher than randomly expected.

### SNP expansion

**Fig. 1:**
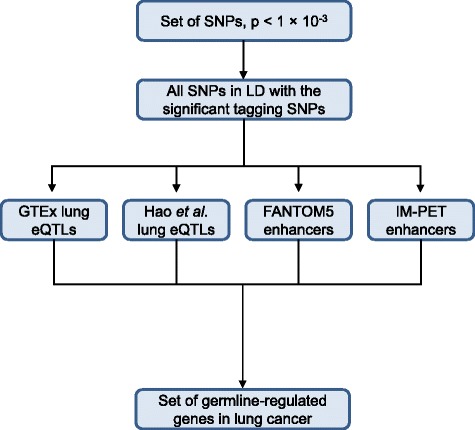
Pipeline to identify germline-regulated genes from the SNPs of the genome-wide association study. The start of our pipeline utilizes moderately associated SNPs (*p* < 1 × 10^− 3^) from the genome-wide association study for three lung cancer subtypes. The outcome is a set of germline-regulated genes in each lung cancer subtype. eQTL expression quantitative trait loci, GTEx Genotype-Tissue Expression project, LD linkage disequilibrium, SNP single-nucleotide polymorphism

To obtain a comprehensive annotation of the SNPs, we expanded our SNP list to include those that are in strong LD with SNPs from the GWAS results at *p* < 1× 10^− 3^. We mapped all SNPs from genome build hg18 to genome build hg19 using the NCBI tool Remap (http://www.ncbi.nlm.nih.gov/genome/tools/remap) and obtained updated SNP rsID numbers using data from dbSNP build 142. We expanded the initial set of genotyped SNPs to include all SNPs in LD within 1 Mb of the genotyped SNP based on data from the European population super group from Phase III of the 1000 Genomes Project [51]. Table 2 outlines the results of this SNP expansion for each subtype. After we removed duplicated SNPs within each subtype, we found 8295 SNPs associated with LUAD, 8734 with LUSC, and 8361 with SCLC, among which 167 SNPs overlapped between all three subtypes (Additional file 1: Figure S2B). We next used the final subtype-specific sets of SNPs from our LD expansion (Table 2) for the subsequent interrogation of regulatory function (Fig. 1).

**Table 2:** Summary of SNP results and LD expansion

	LUAD	LUSC	SCLC
Number of SNPs (GWAS, *p* < 10^− 3^)	544	598	558
Number of SNPs (LD, *r*^2^ 0.8, within 1 Mb)	14,312	16,021	13,104
Number of final SNPs	8295	8734	8361

*LD* linkage disequilibrium, *LUAD* lung adenocarcinoma, *LUSC* lung squamous cell carcinoma, *SCLC* small cell lung cancer, *SNP* single-nucleotide polymorphism

### Lung tissue eQTLs

**Fig. 2:**
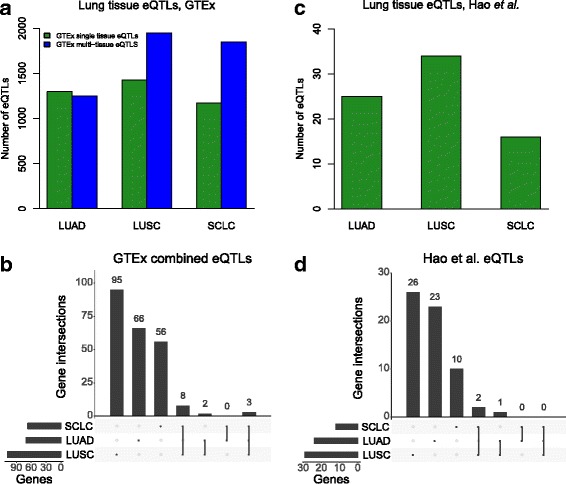
Lung tissue eQTLs in three lung cancer subtypes. **a** Total number of significant eQTLs found in each lung cancer subtype using lung-tissue-specific data (*q* value ≤5%) and multi-tissue data (posterior probability >0.8) from GTEx. **b** UpSetR plot shows the overlap of genes discovered from the GTEx eQTLs. For each lung cancer subtype, we obtained the final gene set by collapsing all SNPs from (**a**) into genes. **c** Total number of eQTLs (false discovery rate < 10%) found in the lung-tissue-specific dataset from Hao et al. [42]. **d** UpSetR plot shows the overlap of genes based on Hao et al. eQTLs. Duplicate genes were removed from **c** for this comparison. eQTL expression quantitative trait loci, GTEx Genotype-Tissue Expression project, LUAD lung adenocarcinoma, LUSC lung squamous cell carcinoma, SCLC small cell lung cancer

We first utilized three lung eQTL datasets to annotate the SNPs. The first lung eQTL dataset was retrieved from the GTEx project [52]. Using this dataset, we found 1297 SNPs for LUAD, 1429 for LUSC, and 1171 for SCLC (Fig. 2a) that acted as eQTLs using a set of pre-compiled significant lung-tissue-specific eQTLs from GTEx. To explore all eQTLs for the lung, including non-tissue-specific eQTLs, we used a second set of eQTLs identified using a multi-tissue model from GTEx (Additional file 1: Figure S1). We combined the single and multi-tissue eQTLs represented by the SNPs to form the final set of GTEx eQTLs. Many of these eQTL SNPs were within strong LD of each other and controlled the expression of the same target gene, so we collapsed all eQTLs to the specific genes they control. As illustrated in Fig. 2b, we found a total of 71 genes for LUAD, 108 for LUSC, and 67 for SCLC. Three genes overlapped from one unique locus in all three subtypes (*CHRNA5, PSMA4,* and *RP11-650 L12.2*). *CHRNA5* is in the nicotinic acetylcholine region that has well-known associations with lung cancer [19, 24, 25] and smoking [53, 54], while *PSMA4* is also associated with lung cancer [55, 56].

We examined a third set of lung tissue eQTLs generated from a meta-analysis that used lung tissue samples from three different recruitment sites (not including GTEx data) [42]. We refer to this eQTL dataset as the Hao et al. eQTLs. We found 25 SNPs for LUAD, 34 for LUSC, and 16 for SCLC that acted as eQTLs (Fig. 2c). We reduced the number of eQTLs to unique target genes (see “Methods”) and found no genes that overlapped all three subtypes, no genes that overlapped LUAD and SCLC, two genes that overlapped LUSC and SCLC in one genomic region (*MYL4* and *RPRML*), and one gene (*IREB2*) that overlapped the two NSCLC subtypes (Fig. 2d). *IREB2* has been previously reported to be associated with both chronic obstructive pulmonary disease and lung cancer, and a recent study suggests a stronger association for lung cancer than chronic obstructive pulmonary disease [57].

### Finding transcribed enhancers and their target genes

**Fig. 3:**
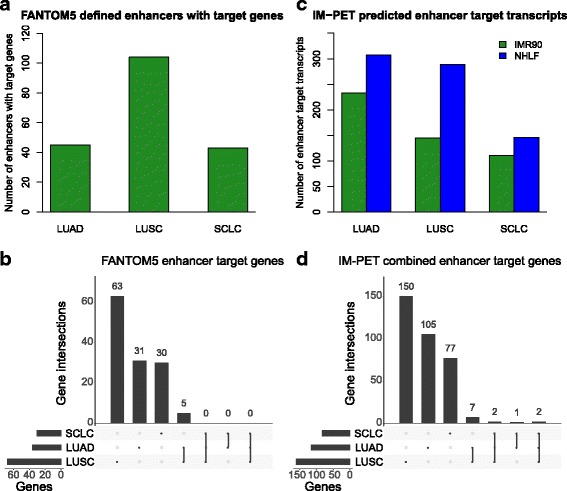
Comparison of the SNPs located within the enhancer regions and their target genes. **a** Total number of enhancer target genes identified by FANTOM5. **b** UpSetR plot shows the overlap of FANTOM5 enhancer target genes by subtype. **c** Total number of enhancer target transcripts identified by IM-PET for two lung-related cell lines. **d** UpSetR plot shows the overlap of the lung cancer predicted enhancer target genes for IMR90 and NHLF identified by IM-PET. LUAD lung adenocarcinoma, LUSC lung squamous cell carcinoma, SCLC small cell lung cancer, SNP single-nucleotide polymorphism

We next examined SNPs located within enhancer regions of the genome that had associated target genes. We used data from the Functional Annotation of the Mammalian Genome (FANTOM) collaborative project [58] that identified transcribed enhancer regions of the genome known as eRNAs using the Cap Analysis of Gene Expression (CAGE) technology [59]. We used this permissive set of enhancers and their corresponding transcribed target genes from the Promoter Enhancer Slider Selector Tool (PrESSTo) website [58, 60]. We found that the number of genes that were targeted by the enhancers was 45 for LUAD, 104 for LUSC, and 43 for SCLC (Fig. 3a). We removed duplicated genes in each subtype and found no overlap for these enhancer target genes among all three subtypes (Fig. 3b). We also observed no overlap among LUAD and SCLC or SCLC and LUSC. However, we did find five target genes from two genomic loci that overlapped LUAD and LUSC (*EPB49, LGI3, LPCAT1, NPM2,* and *PHYHIP*).

### Finding epigenetically defined enhancers and their predicted target genes

To find SNPs located within epigenetically defined enhancers, we used a dataset that defined enhancers using histone modifications such as H3K4me1 [61] and H3K27ac [62]. Specifically, we used the results from a newly developed software tool, IM-PET, that uses specific histone marks to identify enhancers and other data types to predict their targets using a sophisticated random forest classifier [43]. We found more than 100 enhancer targets in all subtypes across two lung-related cell lines (IMR90 and NHLF) (Fig. 3c). These enhancer targets are mRNA transcripts. Therefore, for a comparison similar to that used for the previous datasets, we collapsed all transcripts into single genes (see “Methods”). We merged genes found across both cell lines and removed duplicated genes within subtypes. Only two genes from one unique locus overlapped all subtypes (*ID3HA and TBC1D2B)* (Fig. 3d). *IDH3A* encodes an enzyme in the metabolic tricarboxylic acid (TCA) cycle that is frequently altered in cancer cells [63].

### Final set of germline-regulated genes and comparison to original study

**Fig. 4:**
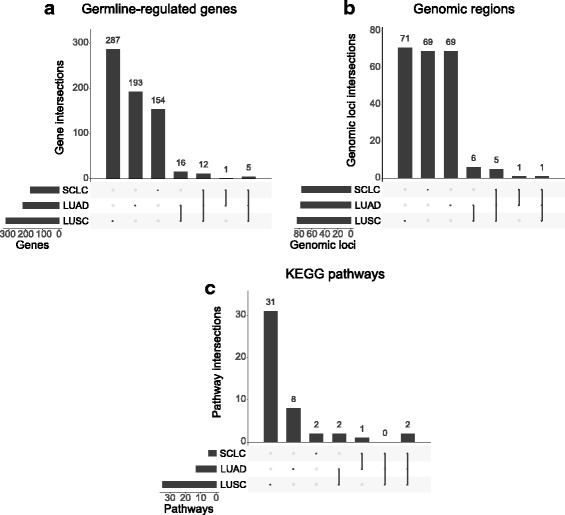
Comparison of the germline-regulated genes, independent genomic loci, and enriched biological pathways by subtype. **a** UpSetR plot shows the overlap of germline-regulated genes identified in the present study for the three lung cancer subtypes. **b** UpSetR plot shows the overlap of independent genomic loci that represent the genes shown in (**a**). **c** UpSetR plot shows the overlap of pathways from the Kyoto Encyclopedia of Genes and Genomes enriched with the germline-regulated genes. KEGG Kyoto Encyclopedia of Genes and Genomes, LUAD lung adenocarcinoma, LUSC lung squamous cell carcinoma, SCLC small cell lung cancer

**Fig. 5:**
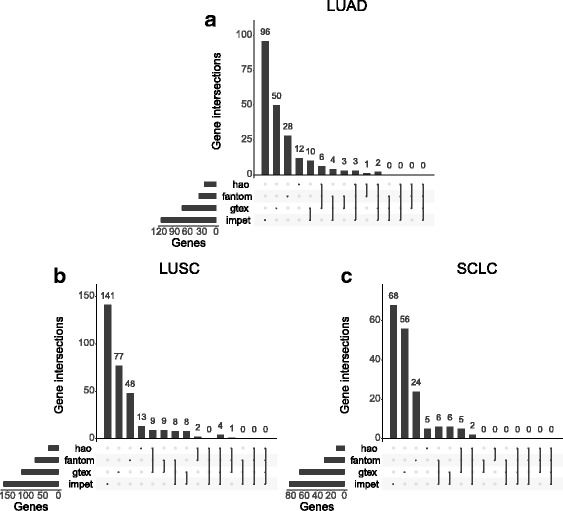
Comparison of the final germline-regulated genes in each subtype separated by the different data sources. UpSetR plots show the overlap between genes found from each data source for **a** lung adenocarcinoma (LUAD), **b** lung squamous cell carcinoma (LUSC), and **c** small cell lung cancer (SCLC). GTEx Genotype-Tissue Expression project, LUAD lung adenocarcinoma, LUSC lung squamous cell carcinoma, SCLC small cell lung cancer

We collected all the genes identified by all of the above methods, removed duplicated genes within subtypes, and referred to this final collection of genes as germline-regulated genes (Additional file 1: Tables S2–S4). Only five genes were shared by all of the subtypes: *CHRNA5*, *IDH3A*, *PSMA4, RP11-650 L12.2,* and *TBC1D2B* (Fig. 4a). Although we found five unique genes, these genes are all located together in one unique genomic region on 15q25 and probably represent only one unique signal. We also compared the genes found across all of the different methods per subtype. Interestingly, we found very little overlap in the target genes identified between the different methods in each subtype. This trend is consistent across all three subtypes (Fig. 5).

A common approach used to report genes that may be associated with SNPs found from a GWAS is to report genes that are the closest in proximity upstream or downstream of the genotyped SNP. Therefore, we next verified that our approach to determine target genes from GWAS SNPs identified a different set of genes than the genes originally reported using the closest gene approach in the original study [20]. For this comparison, we ran the same analysis described above and used the same set of SNPs reported in the original paper’s supplemental tables. We found that only ~25% of the germline-regulated genes that we found using our approach were reported in the original GWAS publication (Additional file 1: Figure S4A).

We further applied our approach to analyze the data from the GWAS Catalog and obtained a set of SNPs for a matched European population type from the GWAS Catalog [64] using the search term “lung cancer” (see “Methods”). After removing the SNPs from our original study, we identified 17 SNPs to run through our pipeline. Additional file 1: Table S5 shows the results from this analysis. We ran the SNPs through the pipeline and identified six germline-regulated genes from the GWAS Catalog SNPs: *CHRNA5, CLPTM1L, PSMA4, RP11-650 L12.2, TP63*, and *ZSCAN29*. We examined the overlap between these genes and our germline-regulated genes by lung cancer subtype in the above subsections. There was a strong overlap (67%) between the genes in at least one subtype from our analysis and the target genes associated with lung cancer from the GWAS Catalog (Additional file 1: Figure S4B).

### Pathway enrichment analysis of germline-regulated genes

To gain a deeper understanding into the biology driven by these germline-regulated genes, we performed biological pathway enrichment analysis of the genes in each subtype. We used the web-based tool, WEB-based Gene Set Analysis Toolkit (WebGestalt) [47], to identify significantly enriched pathways with the set of germline-regulated genes for each subtype using the Kyoto Encyclopedia of Genes and Genomes (KEGG) pathway database. The pathways enriched in each subtype are listed in Additional file 1: Tables S6–S8. We found that all three subtypes had genes enriched in the metabolic pathways and proteasome pathways (Fig. 4c). We note that many of the pathways found for LUSC represent only one genomic locus (HLA region, chromosome 6p21), which contains the same sets of genes (Additional file 1: Table S7).

### Identification of overlapping genes with independent signals

Our approach to identify germline-regulated genes included LD expansion of the preliminary set of GWAS SNPs. Therefore, there were genes that were identified in the same regions of the genome that potentially share the same genetic signal. To determine the number of independent signals we identified in our analysis, we clustered the genes within 1 Mb of each other on each chromosome into a single unique signal. We performed this clustering of genes for all the comparisons that were done at the gene level. We have listed these results for each data source as independent genomic loci in Additional file 1: Tables S9–S15. These results supported the same conclusion from our main analyses.

### Principal component analysis of TCGA germline genotyped SNPs

We also expanded our analysis beyond the GWAS SNPs to determine the degree of genetic sharing using data from TCGA. Importantly, TCGA did not study SCLC, so we were limited to data generated for LUAD and LUSC. We obtained all germline genotyping data from normal blood samples for six cancer types (LUAD, LUSC, head and neck squamous cell carcinoma, bladder urothelial carcinoma, glioblastoma multiforme, and brain lower grade glioma) from TCGA’s data portal (https://portal.gdc.cancer.gov/legacy-archive/search/f). To avoid any genetic influence that may occur due to population differences, we limited our samples to TCGA’s defined white population for our analysis. We ran a PCA on these six cancer types to identify the degree of similarity at the germline genetic level (Additional file 1: Figure S5). Our results indicated that the samples for LUAD and LUSC are no closer to each other spatially than other cancer types. Additionally, several of the samples for LUAD and LUSC are in locations of the plot with other cancer types. These results agree with our regulatory analysis and suggest that LUAD and LUSC do not share much in common with each other at the germline genetic level.

## Discussion

Understanding the genetic risk factors for any cancer type is important in uncovering the underlying biology of the disease. For example, if a SNP is unique to one subtype and acts as an eQTL for a gene involved in a cancer-related pathway, somatic alterations in that gene or other genes in the same pathway can be investigated to understand the development of that subtype. It is also possible that somatic mutations can act in concert with expression-altering SNPs in driving the tumor and would not have the same effect on growth advantage in the absence of the SNP.

Additionally, a specific understanding of the regulatory roles that common genetic variants play in the development of lung cancer subtypes is an important research question because the majority of common variants that increase cancer risk are located within non-coding regions and most likely act as regulators of gene expression. An improved understanding of the carcinogenesis process may provide indications for biomarkers for risk prediction and therapeutic strategies. This is particularly important for SCLC, which is typically diagnosed at a late stage and for which there are not many therapeutic options. To address these questions, we performed a detailed analysis of common genetic variants (SNPs) associated with three subtypes of lung cancer (LUAD, LUSC, and SCLC).

We used marginally significant GWAS results (*p* < 1 × 10^− 3^) to search for regulatory roles for common variants associated with LUAD, LUSC, and SCLC. We expanded this set of results to include all SNPs in LD with the genotyped SNPs using data from the 1000 Genomes Phase III project. This expansion resulted in ~15,000 more SNPs to test per subtype that may be acting as the actual causal variant [65]. We used a diverse set of regulatory data to identify SNPs that were within regulatory regions of the genome that had an identified target gene. Overall, we found a very small overlap between all three subtypes at the SNP, gene, pathway, and regulatory levels. Of note, we found a similar lack of overlap between all subtypes on all levels when we used SNPs with *p* < 1 × 10^− 4^.

It is worth highlighting that three (*CHRNA5, IDH3A,* and *PSMA4)* out of the five genes shared in all three subtypes of lung cancer have been previously reported to be associated with lung cancer. *CHRNA5* has strong implications in its association with lung cancer [19, 24, 25]. *CHRNA5* encodes a nicotinic acetylcholine receptor (nAChR). nAChRs are a class of ligand-gated ion channels that are activated by the neurotransmitter acetylcholine to allow the flow of ions across a cell membrane [66]. There is still an ongoing debate about *CHRNA5*’s role in lung cancer risk versus its risk for lung cancer through nicotine addiction [67], but finding this gene in all three subtypes of lung cancer, which have biological and environmental differences, suggests it may play a direct role in lung cancer risk. *IDH3A* encodes an isocitrate dehydrogenase (IDH). IDHs are important enzymes in the regulation of the TCA cycle [68]. Additionally, *IDH3A* promotes tumor growth by activating hypoxia-inducible factor 1 (HIF-1) and promotes the stability of HIF-1 in participating in angiogenesis and is also associated with poor survival in lung cancer [69]. *IDH3A* also acts in the conversion of metabolism that occurs with cancer fibroblasts [70]. *PSMA4* encodes a subunit of the proteasome*.* Experimental studies have shown that *PSMA4* mRNA is increased in lung tumor versus normal samples and plays major roles in cell proliferation using data from lung carcinoma cell lines [71]. Another gene, *RP11-650 L12.2*, is a non-coding antisense RNA that has not been well characterized. However, one recent study by Jin et al. [72] found a variant in the promoter region of *RP11-650 L12.2* that is associated with risk of colorectal cancer. This finding, in addition to its association with all three subtypes of lung cancer, warrants future experimental studies of this gene. The final gene shared by all subtypes, *TBC1D2B*, is a protein-coding gene that may have GTPase activity and may have a role in autophagy [73].

In addition to the five overlapping genes above, our pathway enrichment analysis revealed two biological pathways shared in the three subtypes. Among them, all three subtypes shared metabolic pathways and the proteasome pathways. Metabolic pathways are frequently modified in cancer to provide the over-proliferating cells with the required nutrients [74, 75]. The proteasome pathway has several links to cell growth in several cancer types [76]. Although these two pathways have a strong relevance to cancer, they are also associated with other disease types due to their components acting in many biological processes. We also observed that the oxidative phosphorylation pathway was significantly enriched in LUSC (Benjamini–Hochberg adjusted *p* = 0.0317). It is interesting to find this pathway dysregulated in the germline genome, because it has strong associations in the transition from oxidative phosphorylation to the less efficient aerobic glycolysis, known as the Warburg effect, which occurs in cancer cell proliferation [77]. Although the Warburg effect may be attributable to glycolysis inhibiting a still active oxidative phosphorylation pathway, this result suggests that commonly occurring variants in LUSC may lead to some disruption in the oxidative phosphorylation pathway that makes this process easier to arrest or inhibit and enhance cell proliferation after some somatic disruption in somatic lung tissue. We also found several cancer-related pathways in LUSC such as pathways in cancer and prostate cancer, and many signaling pathways associated with cancer. We discovered that the focal adhesion pathway was significantly enriched with genes from SCLC (Benjamini–Hochberg adjusted *p* = 0.0275). This is an intriguing finding because this process is involved in the epithelial–mesenchymal transition, which is important in cancer metastasis [78, 79]. In summary, this pathway-based evidence suggests both shared subtype and unique subtype associations.

There are several limitations to this study. First, we utilized a set of marginally significant SNPs. Although previous studies [80, 81] have shown that this is a practical approach, this may have resulted in some false positive SNPs in our study. Second, we did not impute the GWAS data to obtain a larger set of SNPs for the analysis. This would have resulted in more SNPs that could have been tested for significance. We will integrate such SNPs in future analyses. Third, we used the *q* value cutoff identified by GTEx for eQTL significance or non-significance. However, it is possible that there are subtle changes to gene expression from SNPs in the genome and therefore, we may be unintentionally adding or removing SNPs that subtly act in this manner by using a strict predefined cutoff value. Fourth, to validate our results, we were limited to a small set of SNPs reported in the GWAS Catalog because we focused only on SNPs specifically found in one population. While we observed a strong overlap (67%), it would have been better to include a larger set of SNPs for better power of confirming our pipeline. Another limitation of our study is that we may have discovered several different genes that may represent only one unique signal because we used SNPs in LD for our analysis. For example, if we found five genes that were shared by all subtypes, but these genes were clustered in one genomic location, these fives genes may represent only a single unique signal. To account for this potential bias, we separated the genes into unique signals to give a better understanding of the overlap of the subtypes while still including all discovered germline-regulated genes.

## Conclusions

In summary, we used common genetic variants found in three lung cancer subtypes to interrogate the similarity between them at four biological levels (SNP, gene, regulatory, and pathway levels). We found very little overlap between the three subtypes at these levels. At the most basic level (SNPs), we observed less than 1% overlap between the subtypes. Similarly, we found only five genes (from one independent genomic locus) that overlap in all three subtypes, representing <1% of the genes we examined. Three of these five genes (*CHRNA5, IDH3A,* and *PSMA4*) are well-known lung cancer genes. We observed the same trend at the pathway level and found only two KEGG pathways overlapped the subtypes. At the regulatory level, we discovered that many of the enhancer target genes and eQTL target genes are unique to each subtype. Not much work has been done comparing all three subtypes at the somatic level, but recent work interrogating the differences between LUAD and LUSC concluded similarly that there was little overlap between these two subtypes at the molecular level in somatic tumor tissue [18]. Overall, this study provides some important insight into the genetic architecture of three subtypes of lung cancer.

## Additional file

Additional file 1: **Table S1.** Randomization results for overlapping SNPs. **Table S2.** Final germline-regulated genes for LUAD. **Table S3.** Final germline-regulated genes for LUSC. **Table S4.** Final germline-regulated genes for SCLC. **Table S5.** Final germline-regulated genes for GWAS Catalog SNPs. **Table S6.** Pathway enrichment results for LUAD. **Table S7.** Pathway enrichment results for LUSC. **Table S8.** Pathway enrichment results for SCLC. **Table S9.** Independent locus level analysis for genes uniquely identified in LUAD. **Table S10.** Independent locus-level analysis for genes uniquely identified in LUSC. **Table S11.** Independent locus-level analysis for genes uniquely identified in SCLC. **Table S12.** Independent locus-level analysis for LUAD overlap with LUSC. **Table S13.** Independent locus-level analysis for LUAD overlap with SCLC. **Table S14.** Independent locus-level analysis for LUSC overlap with SCLC. **Table S15.** Independent locus-level analysis for all overlaps. **Figure S1.** Determination of significance for GTEx multi-tissue eQTLs. **Figure S2.** Comparison of SNPs from the GWAS for lung cancer. **Figure S3.** Pipelines used to obtain overlap in the LD expanded and LD trimmed SNPs per lung cancer subtype. **Figure S4.** Comparison of germline-regulated genes to original report and the GWAS Catalog. **Figure S5.** PCA of germline genotype data from TCGA in six cancer types. (DOCX 628 kb)

## Abbreviations

eQTL: Expression quantitative trait loci
FANTOM: Functional Annotation of the Mammalian Genome
GTEx: Genotype-Tissue Expression project
GWAS: Genome-wide association study
IM-PET: Integrated Methods for Predicting Enhancer Targets
KEGG: Kyoto Encyclopedia of Genes and Genomes
LD: Linkage disequilibrium
LUAD: Lung adenocarcinoma
LUSC: Lung squamous cell carcinoma
NCBI: National Center for Biotechnology Information
NSCLC: Non-small cell lung cancer
PCA: Principal component analysis
SCLC: Small cell lung cancer
SNP: Single-nucleotide polymorphism
TCGA: The Cancer Genome Atlas
