# Supplementary material for: Weak sharing of genetic association signals in three lung cancer subtypes: evidence at the SNP, gene, regulation, and pathway levels
Source: Genome Med. 2018 Feb 27;10:16. doi: 10.1186/s13073-018-0522-9 (PMC5828003; doi:10.1186/s13073-018-0522-9)
Supplement: Supplementary file 1 — Table S1. Randomization results for overlapping SNPs. Table S2. Final germline-regulated genes for LUAD. Table S3. Final germline-regulated genes for LUSC. Table S4. Final germline-regulated genes for SCLC. Table S5. Final germline-regulated genes for GWAS Catalog SNPs. Table S6. Pathway enrichment results for LUAD. Table S7. Pathway enrichment results for LUSC. Table S8. Pathway enrichment results for SCLC. Table S9. Independent locus level analysis for genes uniquely identified in LUAD. Table S10. Independent locus-level analysis for genes uniquely identified in LUSC. Table S11. Independent locus-level analysis for genes uniquely identified in SCLC. Table S12. Independent locus-level analysis for LUAD overlap with LUSC. Table S13. Independent locus-level analysis for LUAD overlap with SCLC. Table S14. Independent locus-level analysis for LUSC overlap with SCLC. Table S15. Independent locus-level analysis for all overlaps. Figure S1. Determination of significance for GTEx multi-tissue eQTLs. Figure S2. Comparison of SNPs from the GWAS for lung cancer. Figure S3. Pipelines used to obtain overlap in the LD expanded and LD trimmed SNPs per lung cancer subtype. Figure S4. Comparison of germline-regulated genes to original report and the GWAS Catalog. Figure S5. PCA of germline genotype data from TCGA in six cancer types. (DOCX 628 kb) [file 13073_2018_522_MOESM1_ESM.docx]

**Additional file 1**

**Supplemental Data**

**Weak sharing of genetic association signals in three lung cancer subtypes: evidence at the SNP, gene, regulation and pathway levels.**

**Table S1.** Randomization results for overlapping SNPs.

| **Comparison**  **(original SNP overlap) Randomization results of overlapping SNPs out of 10,000 draws** | | | | | | | | | | | |
| --- | --- | --- | --- | --- | --- | --- | --- | --- | --- | --- | --- |
|  | **0** | **1** | **2** | **3** | **4** | **5** | **6** | **7** | **8** | **9** | **10** |
| LUAD overlap LUSC (4) | 5493 | 3281 | 1000 | 198 | 25 | 3 | 0 | 0 | 0 | 0 | 0 |
| LUAD overlap SCLC (1) | 5376 | 3310 | 1046 | 224 | 37 | 7 | 0 | 0 | 0 | 0 | 0 |
| LUSC overlap SCLC (1) | 5465 | 3366 | 949 | 190 | 27 | 2 | 1 | 0 | 0 | 0 | 0 |
| All overlap (1) | 9990 | 10 | 0 | 0 | 0 | 0 | 0 | 0 | 0 | 0 | 0 |

To obtain the values for the table, we randomly selected the same number of SNPs in each subtype 10,000 times and then identified the overlap between those groups of SNPs. This table displays the number of SNPs that overlapped the four comparisons out of 10,000 draws.

**Table S2.** Final germline-regulated genes for LUAD.

| Gene_symbol | Regulatory_cateogry | Data_source |
| --- | --- | --- |
| IGKV1D-13 | Lung specific eQTL | GTEx single tissue V6 Lung eQTL |
| IGKV1-13 | Lung specific eQTL | GTEx single tissue V6 Lung eQTL |
| AC004980.9 | Lung specific eQTL | GTEx single tissue V6 Lung eQTL |
| CHIAP2 | Lung specific eQTL | GTEx single tissue V6 Lung eQTL |
| AF131215.9 | Lung specific eQTL | GTEx single tissue V6 Lung eQTL |
| UNC13D | Lung specific eQTL | GTEx single tissue V6 Lung eQTL |
| RP4-724E13.2 | Lung specific eQTL | GTEx single tissue V6 Lung eQTL |
| RP11-593F23.1 | Lung specific eQTL | GTEx single tissue V6 Lung eQTL |
| CHRNA5 | Lung specific eQTL | GTEx single tissue V6 Lung eQTL |
| RBM6 | Lung specific eQTL | GTEx single tissue V6 Lung eQTL |
| NICN1 | Lung specific eQTL | GTEx single tissue V6 Lung eQTL |
| RNF123 | Lung specific eQTL | GTEx single tissue V6 Lung eQTL |
| IGKV1-17 | Lung specific eQTL | GTEx single tissue V6 Lung eQTL |
| CDKL1 | Lung specific eQTL | GTEx single tissue V6 Lung eQTL |
| MIR5193 | Lung specific eQTL | GTEx single tissue V6 Lung eQTL |
| SPDL1 | Lung specific eQTL | GTEx single tissue V6 Lung eQTL |
| AF131215.2 | Lung specific eQTL | GTEx single tissue V6 Lung eQTL |
| ISL2 | Lung specific eQTL | GTEx single tissue V6 Lung eQTL |
| SECISBP2L | Lung specific eQTL | GTEx single tissue V6 Lung eQTL |
| UGT2B4 | Lung specific eQTL | GTEx single tissue V6 Lung eQTL |
| CLDN23 | Lung specific eQTL | GTEx single tissue V6 Lung eQTL |
| NPHP3 | Lung specific eQTL | GTEx single tissue V6 Lung eQTL |
| AMT | Lung specific eQTL | GTEx single tissue V6 Lung eQTL |
| RP11-62H7.2 | Lung specific eQTL | GTEx single tissue V6 Lung eQTL |
| HOTAIRM1 | Lung specific eQTL | GTEx single tissue V6 Lung eQTL |
| SCAPER | Lung specific eQTL | GTEx single tissue V6 Lung eQTL |
| LGALS9B | Lung specific eQTL | GTEx single tissue V6 Lung eQTL |
| LRRC8C | Lung specific eQTL | GTEx single tissue V6 Lung eQTL |
| RP5-943J3.1 | Lung specific eQTL | GTEx single tissue V6 Lung eQTL |
| RP9P | Lung specific eQTL | GTEx single tissue V6 Lung eQTL |
| RP11-650L12.2 | Lung specific eQTL | GTEx single tissue V6 Lung eQTL |
| AHSA2 | Lung specific eQTL | GTEx single tissue V6 Lung eQTL |
| DDX31 | Lung specific eQTL | GTEx single tissue V6 Lung eQTL |
| COX4I2 | Lung specific eQTL | GTEx single tissue V6 Lung eQTL |
| AC087269.1 | Lung specific eQTL | GTEx single tissue V6 Lung eQTL |
| SLC22A18 | Lung specific eQTL | GTEx single tissue V6 Lung eQTL |
| FBF1 | Lung specific eQTL | GTEx single tissue V6 Lung eQTL |
| DDX11L2 | Lung specific eQTL | GTEx single tissue V6 Lung eQTL |
| RP11-166B2.1 | Lung specific eQTL | GTEx single tissue V6 Lung eQTL |
| DUSP15 | Lung specific eQTL | GTEx single tissue V6 Lung eQTL |
| ENDOD1 | Lung specific eQTL | GTEx single tissue V6 Lung eQTL |
| CHIA | Lung specific eQTL | GTEx single tissue V6 Lung eQTL |
| UBA7 | Lung specific eQTL | GTEx single tissue V6 Lung eQTL |
| CD38 | Lung tissue eQTL | GTEx multi-tissue eQTL in lung > 0.80 |
| RBM6 | Lung tissue eQTL | GTEx multi-tissue eQTL in lung > 0.80 |
| SPDL1 | Lung tissue eQTL | GTEx multi-tissue eQTL in lung > 0.80 |
| PSMA4 | Lung tissue eQTL | GTEx multi-tissue eQTL in lung > 0.80 |
| ULK2 | Lung tissue eQTL | GTEx multi-tissue eQTL in lung > 0.80 |
| MLF2 | Lung tissue eQTL | GTEx multi-tissue eQTL in lung > 0.80 |
| UNC13D | Lung tissue eQTL | GTEx multi-tissue eQTL in lung > 0.80 |
| CDKL1 | Lung tissue eQTL | GTEx multi-tissue eQTL in lung > 0.80 |
| CTSH | Lung tissue eQTL | GTEx multi-tissue eQTL in lung > 0.80 |
| ZNF175 | Lung tissue eQTL | GTEx multi-tissue eQTL in lung > 0.80 |
| HOXA2 | Lung tissue eQTL | GTEx multi-tissue eQTL in lung > 0.80 |
| CWC25 | Lung tissue eQTL | GTEx multi-tissue eQTL in lung > 0.80 |
| AKAP10 | Lung tissue eQTL | GTEx multi-tissue eQTL in lung > 0.80 |
| SLC22A18 | Lung tissue eQTL | GTEx multi-tissue eQTL in lung > 0.80 |
| NPHP3 | Lung tissue eQTL | GTEx multi-tissue eQTL in lung > 0.80 |
| HOXA7 | Lung tissue eQTL | GTEx multi-tissue eQTL in lung > 0.80 |
| DDX31 | Lung tissue eQTL | GTEx multi-tissue eQTL in lung > 0.80 |
| COX4I2 | Lung tissue eQTL | GTEx multi-tissue eQTL in lung > 0.80 |
| ADAMTS7 | Lung tissue eQTL | GTEx multi-tissue eQTL in lung > 0.80 |
| SECISBP2L | Lung tissue eQTL | GTEx multi-tissue eQTL in lung > 0.80 |
| ETFA | Lung tissue eQTL | GTEx multi-tissue eQTL in lung > 0.80 |
| TRIM65 | Lung tissue eQTL | GTEx multi-tissue eQTL in lung > 0.80 |
| ZCCHC2 | Lung tissue eQTL | GTEx multi-tissue eQTL in lung > 0.80 |
| PIP4K2B | Lung tissue eQTL | GTEx multi-tissue eQTL in lung > 0.80 |
| ENDOD1 | Lung tissue eQTL | GTEx multi-tissue eQTL in lung > 0.80 |
| DUSP15 | Lung tissue eQTL | GTEx multi-tissue eQTL in lung > 0.80 |
| ZFYVE28 | Lung tissue eQTL | GTEx multi-tissue eQTL in lung > 0.80 |
| PPAPDC3 | Lung tissue eQTL | GTEx multi-tissue eQTL in lung > 0.80 |
| RNF123 | Lung tissue eQTL | GTEx multi-tissue eQTL in lung > 0.80 |
| MST1R | Lung tissue eQTL | GTEx multi-tissue eQTL in lung > 0.80 |
| TSC1 | Lung tissue eQTL | GTEx multi-tissue eQTL in lung > 0.80 |
| CHRNA5 | Lung tissue eQTL | GTEx multi-tissue eQTL in lung > 0.80 |
| AHSA2 | Lung tissue eQTL | GTEx multi-tissue eQTL in lung > 0.80 |
| ZNF266 | Lung tissue eQTL | GTEx multi-tissue eQTL in lung > 0.80 |
| KBTBD11 | Lung tissue eQTL | GTEx multi-tissue eQTL in lung > 0.80 |
| DEFB124 | Lung tissue eQTL | GTEx multi-tissue eQTL in lung > 0.80 |
| UBA7 | Lung tissue eQTL | GTEx multi-tissue eQTL in lung > 0.80 |
| FBF1 | Lung tissue eQTL | GTEx multi-tissue eQTL in lung > 0.80 |
| TMEM184B | Lung tissue eQTL | GTEx multi-tissue eQTL in lung > 0.80 |
| RP5-943J3.1 | Lung tissue eQTL | GTEx multi-tissue eQTL in lung > 0.80 |
| CTD-2012J19.1 | Lung tissue eQTL | GTEx multi-tissue eQTL in lung > 0.80 |
| HOTAIRM1 | Lung tissue eQTL | GTEx multi-tissue eQTL in lung > 0.80 |
| RP11-166B2.1 | Lung tissue eQTL | GTEx multi-tissue eQTL in lung > 0.80 |
| RP11-108M9.5 | Lung tissue eQTL | GTEx multi-tissue eQTL in lung > 0.80 |
| HOXA10 | Lung tissue eQTL | GTEx multi-tissue eQTL in lung > 0.80 |
| AL021977.1 | Lung tissue eQTL | GTEx multi-tissue eQTL in lung > 0.80 |
| RP11-166B2.7 | Lung tissue eQTL | GTEx multi-tissue eQTL in lung > 0.80 |
| RP11-650L12.2 | Lung tissue eQTL | GTEx multi-tissue eQTL in lung > 0.80 |
| ASAP1 | Lung specific eQTL | Hao *et al*. single tissue lung eQTL |
| LYAR | Lung specific eQTL | Hao *et al*. single tissue lung eQTL |
| SLC2A5 | Lung specific eQTL | Hao *et al*. single tissue lung eQTL |
| RBM6 | Lung specific eQTL | Hao *et al*. single tissue lung eQTL |
| COX4I2 | Lung specific eQTL | Hao *et al*. single tissue lung eQTL |
| UNC13D | Lung specific eQTL | Hao *et al*. single tissue lung eQTL |
| UNK | Lung specific eQTL | Hao *et al*. single tissue lung eQTL |
| CHIA | Lung specific eQTL | Hao *et al*. single tissue lung eQTL |
| IREB2 | Lung specific eQTL | Hao *et al*. single tissue lung eQTL |
| SFN | Lung specific eQTL | Hao *et al*. single tissue lung eQTL |
| SLC9A1 | Lung specific eQTL | Hao *et al*. single tissue lung eQTL |
| SECISBP2L | Lung specific eQTL | Hao *et al*. single tissue lung eQTL |
| PEX13 | Lung specific eQTL | Hao *et al*. single tissue lung eQTL |
| ETFA | Lung specific eQTL | Hao *et al*. single tissue lung eQTL |
| ACOX1 | Lung specific eQTL | Hao *et al*. single tissue lung eQTL |
| CDKL1 | Lung specific eQTL | Hao *et al*. single tissue lung eQTL |
| BCL2L1 | Lung specific eQTL | Hao *et al*. single tissue lung eQTL |
| CHI3L2 | Lung specific eQTL | Hao *et al*. single tissue lung eQTL |
| FLJ27354 | Lung specific eQTL | Hao *et al*. single tissue lung eQTL |
| ENDOD1 | Lung specific eQTL | Hao *et al*. single tissue lung eQTL |
| HNRNPA2B1 | Lung specific eQTL | Hao *et al*. single tissue lung eQTL |
| NCAM1 | Lung specific eQTL | Hao *et al*. single tissue lung eQTL |
| WDFY2 | Lung specific eQTL | Hao *et al*. single tissue lung eQTL |
| UBA5 | Lung specific eQTL | Hao *et al*. single tissue lung eQTL |
| PADI4 | permissive enhancer | FANTOM5 enhancer target gene |
| PADI2 | permissive enhancer | FANTOM5 enhancer target gene |
| LPCAT1 | permissive enhancer | FANTOM5 enhancer target gene |
| LIN28B | permissive enhancer | FANTOM5 enhancer target gene |
| HACE1 | permissive enhancer | FANTOM5 enhancer target gene |
| C7orf31 | permissive enhancer | FANTOM5 enhancer target gene |
| DFNA5 | permissive enhancer | FANTOM5 enhancer target gene |
| PHYHIP | permissive enhancer | FANTOM5 enhancer target gene |
| KBTBD11 | permissive enhancer | FANTOM5 enhancer target gene |
| LGI3 | permissive enhancer | FANTOM5 enhancer target gene |
| EPB49 | permissive enhancer | FANTOM5 enhancer target gene |
| NPM2 | permissive enhancer | FANTOM5 enhancer target gene |
| AKAP2 | permissive enhancer | FANTOM5 enhancer target gene |
| GSTO1 | permissive enhancer | FANTOM5 enhancer target gene |
| SLK | permissive enhancer | FANTOM5 enhancer target gene |
| KCNA1 | permissive enhancer | FANTOM5 enhancer target gene |
| PPM1H | permissive enhancer | FANTOM5 enhancer target gene |
| KCNA6 | permissive enhancer | FANTOM5 enhancer target gene |
| C13orf15 | permissive enhancer | FANTOM5 enhancer target gene |
| FOXO1 | permissive enhancer | FANTOM5 enhancer target gene |
| NAA16 | permissive enhancer | FANTOM5 enhancer target gene |
| ELF1 | permissive enhancer | FANTOM5 enhancer target gene |
| IREB2 | permissive enhancer | FANTOM5 enhancer target gene |
| MORF4L1 | permissive enhancer | FANTOM5 enhancer target gene |
| MYH4 | permissive enhancer | FANTOM5 enhancer target gene |
| MAPK7 | permissive enhancer | FANTOM5 enhancer target gene |
| NEDD4L | permissive enhancer | FANTOM5 enhancer target gene |
| KIAA1468 | permissive enhancer | FANTOM5 enhancer target gene |
| ZCCHC2 | permissive enhancer | FANTOM5 enhancer target gene |
| PHLPP1 | permissive enhancer | FANTOM5 enhancer target gene |
| TTLL9 | permissive enhancer | FANTOM5 enhancer target gene |
| DUSP15 | permissive enhancer | FANTOM5 enhancer target gene |
| DEFB116 | permissive enhancer | FANTOM5 enhancer target gene |
| DEFB121 | permissive enhancer | FANTOM5 enhancer target gene |
| HM13 | permissive enhancer | FANTOM5 enhancer target gene |
| ID1 | permissive enhancer | FANTOM5 enhancer target gene |
| UNC13D | predicted enhancer target | IM-PET IMR90 |
| HOXA5 | predicted enhancer target | IM-PET IMR90 |
| HOXA6 | predicted enhancer target | IM-PET IMR90 |
| HOXA7 | predicted enhancer target | IM-PET IMR90 |
| INHBA | predicted enhancer target | IM-PET IMR90 |
| CEP89 | predicted enhancer target | IM-PET IMR90 |
| KLF7 | predicted enhancer target | IM-PET IMR90 |
| CHMP6 | predicted enhancer target | IM-PET IMR90 |
| TPX2 | predicted enhancer target | IM-PET IMR90 |
| HM13 | predicted enhancer target | IM-PET IMR90 |
| HOXA1 | predicted enhancer target | IM-PET IMR90 |
| LLGL2 | predicted enhancer target | IM-PET IMR90 |
| C20orf112 | predicted enhancer target | IM-PET IMR90 |
| ID1 | predicted enhancer target | IM-PET IMR90 |
| CTD-3035D6.1 | predicted enhancer target | IM-PET IMR90 |
| HOXA3 | predicted enhancer target | IM-PET IMR90 |
| SHC4 | predicted enhancer target | IM-PET IMR90 |
| TM9SF4 | predicted enhancer target | IM-PET IMR90 |
| PIGN | predicted enhancer target | IM-PET IMR90 |
| IFRD2 | predicted enhancer target | IM-PET IMR90 |
| BCL2L1 | predicted enhancer target | IM-PET IMR90 |
| NT5C2 | predicted enhancer target | IM-PET IMR90 |
| RP11-108M9.4 | predicted enhancer target | IM-PET IMR90 |
| MDH2 | predicted enhancer target | IM-PET IMR90 |
| MORF4L1 | predicted enhancer target | IM-PET IMR90 |
| HOTAIRM1 | predicted enhancer target | IM-PET IMR90 |
| TGM4 | predicted enhancer target | IM-PET IMR90 |
| QARS | predicted enhancer target | IM-PET IMR90 |
| AKAP10 | predicted enhancer target | IM-PET IMR90 |
| POR | predicted enhancer target | IM-PET IMR90 |
| IMPDH2 | predicted enhancer target | IM-PET IMR90 |
| OBFC1 | predicted enhancer target | IM-PET IMR90 |
| RP1-170O19.22 | predicted enhancer target | IM-PET IMR90 |
| IP6K1 | predicted enhancer target | IM-PET IMR90 |
| UBA5 | predicted enhancer target | IM-PET IMR90 |
| WDR6 | predicted enhancer target | IM-PET IMR90 |
| CALHM2 | predicted enhancer target | IM-PET IMR90 |
| RNF123 | predicted enhancer target | IM-PET IMR90 |
| RP1-170O19.23 | predicted enhancer target | IM-PET IMR90 |
| ALS2CL | predicted enhancer target | IM-PET IMR90 |
| HOXA-AS2 | predicted enhancer target | IM-PET IMR90 |
| ASAP1 | predicted enhancer target | IM-PET IMR90 |
| XPO7 | predicted enhancer target | IM-PET IMR90 |
| HOXA-AS3 | predicted enhancer target | IM-PET IMR90 |
| GGA3 | predicted enhancer target | IM-PET IMR90 |
| HOXA11 | predicted enhancer target | IM-PET NHLF |
| TRIB2 | predicted enhancer target | IM-PET NHLF |
| MYH9 | predicted enhancer target | IM-PET NHLF |
| CAV2 | predicted enhancer target | IM-PET NHLF |
| HOXA2 | predicted enhancer target | IM-PET NHLF |
| HOXA5 | predicted enhancer target | IM-PET NHLF |
| HOXA7 | predicted enhancer target | IM-PET NHLF |
| RPL23 | predicted enhancer target | IM-PET NHLF |
| NECAB3 | predicted enhancer target | IM-PET NHLF |
| PLAGL2 | predicted enhancer target | IM-PET NHLF |
| C1QTNF6 | predicted enhancer target | IM-PET NHLF |
| HIBADH | predicted enhancer target | IM-PET NHLF |
| HOXA10 | predicted enhancer target | IM-PET NHLF |
| IDH3A | predicted enhancer target | IM-PET NHLF |
| ASXL1 | predicted enhancer target | IM-PET NHLF |
| SLC16A6 | predicted enhancer target | IM-PET NHLF |
| FAM212A | predicted enhancer target | IM-PET NHLF |
| UBA7 | predicted enhancer target | IM-PET NHLF |
| HOXA1 | predicted enhancer target | IM-PET NHLF |
| ZNF384 | predicted enhancer target | IM-PET NHLF |
| SH3PXD2A | predicted enhancer target | IM-PET NHLF |
| PRKAR1A | predicted enhancer target | IM-PET NHLF |
| NID1 | predicted enhancer target | IM-PET NHLF |
| CALHM2 | predicted enhancer target | IM-PET NHLF |
| SUFU | predicted enhancer target | IM-PET NHLF |
| GBF1 | predicted enhancer target | IM-PET NHLF |
| TMEM51 | predicted enhancer target | IM-PET NHLF |
| ID1 | predicted enhancer target | IM-PET NHLF |
| SECISBP2L | predicted enhancer target | IM-PET NHLF |
| ST7 | predicted enhancer target | IM-PET NHLF |
| CAV1 | predicted enhancer target | IM-PET NHLF |
| GAPDH | predicted enhancer target | IM-PET NHLF |
| CARS | predicted enhancer target | IM-PET NHLF |
| TM9SF4 | predicted enhancer target | IM-PET NHLF |
| HM13 | predicted enhancer target | IM-PET NHLF |
| KIAA2026 | predicted enhancer target | IM-PET NHLF |
| PHLPP1 | predicted enhancer target | IM-PET NHLF |
| TMEM184B | predicted enhancer target | IM-PET NHLF |
| PSMD14 | predicted enhancer target | IM-PET NHLF |
| USP34 | predicted enhancer target | IM-PET NHLF |
| CDHR4 | predicted enhancer target | IM-PET NHLF |
| ANKRD54 | predicted enhancer target | IM-PET NHLF |
| JAZF1 | predicted enhancer target | IM-PET NHLF |
| TANK | predicted enhancer target | IM-PET NHLF |
| GGA1 | predicted enhancer target | IM-PET NHLF |
| TAX1BP1 | predicted enhancer target | IM-PET NHLF |
| RP11-160C18.4 | predicted enhancer target | IM-PET NHLF |
| BCL2L1 | predicted enhancer target | IM-PET NHLF |
| INHBA | predicted enhancer target | IM-PET NHLF |
| AC037459.4 | predicted enhancer target | IM-PET NHLF |
| TBC1D2B | predicted enhancer target | IM-PET NHLF |
| TES | predicted enhancer target | IM-PET NHLF |
| RBFOX2 | predicted enhancer target | IM-PET NHLF |
| LGALS1 | predicted enhancer target | IM-PET NHLF |
| CROCC | predicted enhancer target | IM-PET NHLF |
| OBFC1 | predicted enhancer target | IM-PET NHLF |
| NT5C2 | predicted enhancer target | IM-PET NHLF |
| RHBDD2 | predicted enhancer target | IM-PET NHLF |
| RNF123 | predicted enhancer target | IM-PET NHLF |
| CAMKV | predicted enhancer target | IM-PET NHLF |
| MRPL38 | predicted enhancer target | IM-PET NHLF |
| MST1 | predicted enhancer target | IM-PET NHLF |
| SKAP2 | predicted enhancer target | IM-PET NHLF |
| QARS | predicted enhancer target | IM-PET NHLF |
| DNAJC13 | predicted enhancer target | IM-PET NHLF |
| HOXA9 | predicted enhancer target | IM-PET NHLF |
| GGPS1 | predicted enhancer target | IM-PET NHLF |
| UGCG | predicted enhancer target | IM-PET NHLF |
| CAPZA2 | predicted enhancer target | IM-PET NHLF |
| PCGF6 | predicted enhancer target | IM-PET NHLF |
| IP6K1 | predicted enhancer target | IM-PET NHLF |
| LAMB2 | predicted enhancer target | IM-PET NHLF |
| RP1-170O19.23 | predicted enhancer target | IM-PET NHLF |
| ASAP1 | predicted enhancer target | IM-PET NHLF |
| POLR3D | predicted enhancer target | IM-PET NHLF |
| EFR3A | predicted enhancer target | IM-PET NHLF |
| HOXA6 | predicted enhancer target | IM-PET NHLF |
| HOXA3 | predicted enhancer target | IM-PET NHLF |
| OSBPL5 | predicted enhancer target | IM-PET NHLF |
| CTSH | predicted enhancer target | IM-PET NHLF |
| GPR68 | predicted enhancer target | IM-PET NHLF |
| PIANP | predicted enhancer target | IM-PET NHLF |
| AMIGO3 | predicted enhancer target | IM-PET NHLF |
| MLF2 | predicted enhancer target | IM-PET NHLF |
| CDCA3 | predicted enhancer target | IM-PET NHLF |
| CLSTN3 | predicted enhancer target | IM-PET NHLF |
| GNB3 | predicted enhancer target | IM-PET NHLF |
| WIPI1 | predicted enhancer target | IM-PET NHLF |

**Table S3.** Final germline-regulated genes for LUSC.

| Gene_symbol | Regulatory_cateogry | Data_source |
| --- | --- | --- |
| PMM2 | Lung specific eQTL | GTEx single tissue V6 Lung eQTL |
| TCTEX1D1 | Lung specific eQTL | GTEx single tissue V6 Lung eQTL |
| NFATC3 | Lung specific eQTL | GTEx single tissue V6 Lung eQTL |
| HLA-DQA1 | Lung specific eQTL | GTEx single tissue V6 Lung eQTL |
| HLA-DPA1 | Lung specific eQTL | GTEx single tissue V6 Lung eQTL |
| XXbac-BPG248L24.12 | Lung specific eQTL | GTEx single tissue V6 Lung eQTL |
| PIGZ | Lung specific eQTL | GTEx single tissue V6 Lung eQTL |
| MYL4 | Lung specific eQTL | GTEx single tissue V6 Lung eQTL |
| ZMAT2 | Lung specific eQTL | GTEx single tissue V6 Lung eQTL |
| DCAKD | Lung specific eQTL | GTEx single tissue V6 Lung eQTL |
| RFTN2 | Lung specific eQTL | GTEx single tissue V6 Lung eQTL |
| PCDHGA5 | Lung specific eQTL | GTEx single tissue V6 Lung eQTL |
| CHRNA5 | Lung specific eQTL | GTEx single tissue V6 Lung eQTL |
| PON1 | Lung specific eQTL | GTEx single tissue V6 Lung eQTL |
| AVEN | Lung specific eQTL | GTEx single tissue V6 Lung eQTL |
| HCG4P5 | Lung specific eQTL | GTEx single tissue V6 Lung eQTL |
| HFM1 | Lung specific eQTL | GTEx single tissue V6 Lung eQTL |
| CTD-2265M8.2 | Lung specific eQTL | GTEx single tissue V6 Lung eQTL |
| HCG23 | Lung specific eQTL | GTEx single tissue V6 Lung eQTL |
| DCTN5 | Lung specific eQTL | GTEx single tissue V6 Lung eQTL |
| HLA-DRB5 | Lung specific eQTL | GTEx single tissue V6 Lung eQTL |
| CTD-2083E4.5 | Lung specific eQTL | GTEx single tissue V6 Lung eQTL |
| RWDD3 | Lung specific eQTL | GTEx single tissue V6 Lung eQTL |
| PON3 | Lung specific eQTL | GTEx single tissue V6 Lung eQTL |
| RP3-414A15.2 | Lung specific eQTL | GTEx single tissue V6 Lung eQTL |
| HLA-J | Lung specific eQTL | GTEx single tissue V6 Lung eQTL |
| HLA-DQB1 | Lung specific eQTL | GTEx single tissue V6 Lung eQTL |
| PCDHA10 | Lung specific eQTL | GTEx single tissue V6 Lung eQTL |
| TMCO6 | Lung specific eQTL | GTEx single tissue V6 Lung eQTL |
| RP11-463O9.9 | Lung specific eQTL | GTEx single tissue V6 Lung eQTL |
| HLA-DRB1 | Lung specific eQTL | GTEx single tissue V6 Lung eQTL |
| AC007308.7 | Lung specific eQTL | GTEx single tissue V6 Lung eQTL |
| RP11-137H2.4 | Lung specific eQTL | GTEx single tissue V6 Lung eQTL |
| PCDHB16 | Lung specific eQTL | GTEx single tissue V6 Lung eQTL |
| NSUN2 | Lung specific eQTL | GTEx single tissue V6 Lung eQTL |
| HEATR4 | Lung specific eQTL | GTEx single tissue V6 Lung eQTL |
| CELF2 | Lung specific eQTL | GTEx single tissue V6 Lung eQTL |
| SKIV2L | Lung specific eQTL | GTEx single tissue V6 Lung eQTL |
| RP11-650L12.2 | Lung specific eQTL | GTEx single tissue V6 Lung eQTL |
| HLA-K | Lung specific eQTL | GTEx single tissue V6 Lung eQTL |
| TSC1 | Lung specific eQTL | GTEx single tissue V6 Lung eQTL |
| PCDHA7 | Lung specific eQTL | GTEx single tissue V6 Lung eQTL |
| PRMT7 | Lung specific eQTL | GTEx single tissue V6 Lung eQTL |
| PON2 | Lung specific eQTL | GTEx single tissue V6 Lung eQTL |
| PLEKHG4B | Lung specific eQTL | GTEx single tissue V6 Lung eQTL |
| GS1-293C5.1 | Lung specific eQTL | GTEx single tissue V6 Lung eQTL |
| ZFP57 | Lung specific eQTL | GTEx single tissue V6 Lung eQTL |
| HLA-DRB6 | Lung specific eQTL | GTEx single tissue V6 Lung eQTL |
| PLA2G4C | Lung specific eQTL | GTEx single tissue V6 Lung eQTL |
| HLA-DOB | Lung specific eQTL | GTEx single tissue V6 Lung eQTL |
| XXbac-BPG254F23.6 | Lung specific eQTL | GTEx single tissue V6 Lung eQTL |
| DCAF4 | Lung specific eQTL | GTEx single tissue V6 Lung eQTL |
| ACOT2 | Lung specific eQTL | GTEx single tissue V6 Lung eQTL |
| RAD52 | Lung tissue eQTL | GTEx multi-tissue eQTL in lung > 0.80 |
| NSUN2 | Lung tissue eQTL | GTEx multi-tissue eQTL in lung > 0.80 |
| PSMA4 | Lung tissue eQTL | GTEx multi-tissue eQTL in lung > 0.80 |
| AHRR | Lung tissue eQTL | GTEx multi-tissue eQTL in lung > 0.80 |
| SDHA | Lung tissue eQTL | GTEx multi-tissue eQTL in lung > 0.80 |
| MUL1 | Lung tissue eQTL | GTEx multi-tissue eQTL in lung > 0.80 |
| NLRC4 | Lung tissue eQTL | GTEx multi-tissue eQTL in lung > 0.80 |
| MTHFSD | Lung tissue eQTL | GTEx multi-tissue eQTL in lung > 0.80 |
| EARS2 | Lung tissue eQTL | GTEx multi-tissue eQTL in lung > 0.80 |
| PLA2G4C | Lung tissue eQTL | GTEx multi-tissue eQTL in lung > 0.80 |
| PON2 | Lung tissue eQTL | GTEx multi-tissue eQTL in lung > 0.80 |
| CORO2A | Lung tissue eQTL | GTEx multi-tissue eQTL in lung > 0.80 |
| SLC22A18 | Lung tissue eQTL | GTEx multi-tissue eQTL in lung > 0.80 |
| TMCO6 | Lung tissue eQTL | GTEx multi-tissue eQTL in lung > 0.80 |
| IK | Lung tissue eQTL | GTEx multi-tissue eQTL in lung > 0.80 |
| EDEM3 | Lung tissue eQTL | GTEx multi-tissue eQTL in lung > 0.80 |
| PIGZ | Lung tissue eQTL | GTEx multi-tissue eQTL in lung > 0.80 |
| DCAF4 | Lung tissue eQTL | GTEx multi-tissue eQTL in lung > 0.80 |
| ACOT2 | Lung tissue eQTL | GTEx multi-tissue eQTL in lung > 0.80 |
| WDR55 | Lung tissue eQTL | GTEx multi-tissue eQTL in lung > 0.80 |
| RWDD3 | Lung tissue eQTL | GTEx multi-tissue eQTL in lung > 0.80 |
| OPN1SW | Lung tissue eQTL | GTEx multi-tissue eQTL in lung > 0.80 |
| DCAF8 | Lung tissue eQTL | GTEx multi-tissue eQTL in lung > 0.80 |
| STAB2 | Lung tissue eQTL | GTEx multi-tissue eQTL in lung > 0.80 |
| USP44 | Lung tissue eQTL | GTEx multi-tissue eQTL in lung > 0.80 |
| PPIL1 | Lung tissue eQTL | GTEx multi-tissue eQTL in lung > 0.80 |
| PMM2 | Lung tissue eQTL | GTEx multi-tissue eQTL in lung > 0.80 |
| SRD5A1 | Lung tissue eQTL | GTEx multi-tissue eQTL in lung > 0.80 |
| CYP17A1 | Lung tissue eQTL | GTEx multi-tissue eQTL in lung > 0.80 |
| LIN7C | Lung tissue eQTL | GTEx multi-tissue eQTL in lung > 0.80 |
| TCTEX1D1 | Lung tissue eQTL | GTEx multi-tissue eQTL in lung > 0.80 |
| CARHSP1 | Lung tissue eQTL | GTEx multi-tissue eQTL in lung > 0.80 |
| FAM69A | Lung tissue eQTL | GTEx multi-tissue eQTL in lung > 0.80 |
| CCDC127 | Lung tissue eQTL | GTEx multi-tissue eQTL in lung > 0.80 |
| TSC1 | Lung tissue eQTL | GTEx multi-tissue eQTL in lung > 0.80 |
| C10orf32 | Lung tissue eQTL | GTEx multi-tissue eQTL in lung > 0.80 |
| DCTN5 | Lung tissue eQTL | GTEx multi-tissue eQTL in lung > 0.80 |
| DUSP18 | Lung tissue eQTL | GTEx multi-tissue eQTL in lung > 0.80 |
| CHRNA5 | Lung tissue eQTL | GTEx multi-tissue eQTL in lung > 0.80 |
| AVEN | Lung tissue eQTL | GTEx multi-tissue eQTL in lung > 0.80 |
| FEZ2 | Lung tissue eQTL | GTEx multi-tissue eQTL in lung > 0.80 |
| RHNO1 | Lung tissue eQTL | GTEx multi-tissue eQTL in lung > 0.80 |
| ZNF131 | Lung tissue eQTL | GTEx multi-tissue eQTL in lung > 0.80 |
| DCAKD | Lung tissue eQTL | GTEx multi-tissue eQTL in lung > 0.80 |
| HLA-DQB1 | Lung tissue eQTL | GTEx multi-tissue eQTL in lung > 0.80 |
| RPRML | Lung tissue eQTL | GTEx multi-tissue eQTL in lung > 0.80 |
| ACOT1 | Lung tissue eQTL | GTEx multi-tissue eQTL in lung > 0.80 |
| TMEM186 | Lung tissue eQTL | GTEx multi-tissue eQTL in lung > 0.80 |
| CHRM5 | Lung tissue eQTL | GTEx multi-tissue eQTL in lung > 0.80 |
| HEATR4 | Lung tissue eQTL | GTEx multi-tissue eQTL in lung > 0.80 |
| HLA-DRB1 | Lung tissue eQTL | GTEx multi-tissue eQTL in lung > 0.80 |
| HLA-DQA1 | Lung tissue eQTL | GTEx multi-tissue eQTL in lung > 0.80 |
| SKIV2L | Lung tissue eQTL | GTEx multi-tissue eQTL in lung > 0.80 |
| NELFE | Lung tissue eQTL | GTEx multi-tissue eQTL in lung > 0.80 |
| MICB | Lung tissue eQTL | GTEx multi-tissue eQTL in lung > 0.80 |
| RP11-345J4.8 | Lung tissue eQTL | GTEx multi-tissue eQTL in lung > 0.80 |
| SRA1 | Lung tissue eQTL | GTEx multi-tissue eQTL in lung > 0.80 |
| AS3MT | Lung tissue eQTL | GTEx multi-tissue eQTL in lung > 0.80 |
| C5orf55 | Lung tissue eQTL | GTEx multi-tissue eQTL in lung > 0.80 |
| HLA-DQB1-AS1 | Lung tissue eQTL | GTEx multi-tissue eQTL in lung > 0.80 |
| RP11-137H2.4 | Lung tissue eQTL | GTEx multi-tissue eQTL in lung > 0.80 |
| HLA-DRB6 | Lung tissue eQTL | GTEx multi-tissue eQTL in lung > 0.80 |
| SSR4P1 | Lung tissue eQTL | GTEx multi-tissue eQTL in lung > 0.80 |
| HLA-DQA2 | Lung tissue eQTL | GTEx multi-tissue eQTL in lung > 0.80 |
| XXbac-BPG254F23.6 | Lung tissue eQTL | GTEx multi-tissue eQTL in lung > 0.80 |
| EIF4EBP3 | Lung tissue eQTL | GTEx multi-tissue eQTL in lung > 0.80 |
| RN7SL834P | Lung tissue eQTL | GTEx multi-tissue eQTL in lung > 0.80 |
| RP11-463O9.1 | Lung tissue eQTL | GTEx multi-tissue eQTL in lung > 0.80 |
| TNXA | Lung tissue eQTL | GTEx multi-tissue eQTL in lung > 0.80 |
| CTD-2083E4.6 | Lung tissue eQTL | GTEx multi-tissue eQTL in lung > 0.80 |
| KRT8P46 | Lung tissue eQTL | GTEx multi-tissue eQTL in lung > 0.80 |
| PDCD6 | Lung tissue eQTL | GTEx multi-tissue eQTL in lung > 0.80 |
| LINC01018 | Lung tissue eQTL | GTEx multi-tissue eQTL in lung > 0.80 |
| STK19P | Lung tissue eQTL | GTEx multi-tissue eQTL in lung > 0.80 |
| CTD-2083E4.5 | Lung tissue eQTL | GTEx multi-tissue eQTL in lung > 0.80 |
| RP3-414A15.2 | Lung tissue eQTL | GTEx multi-tissue eQTL in lung > 0.80 |
| RP11-109N23.6 | Lung tissue eQTL | GTEx multi-tissue eQTL in lung > 0.80 |
| RP11-152P23.2 | Lung tissue eQTL | GTEx multi-tissue eQTL in lung > 0.80 |
| RP11-650L12.2 | Lung tissue eQTL | GTEx multi-tissue eQTL in lung > 0.80 |
| RP11-156P1.3 | Lung tissue eQTL | GTEx multi-tissue eQTL in lung > 0.80 |
| LRRC37A17P | Lung tissue eQTL | GTEx multi-tissue eQTL in lung > 0.80 |
| TMCO6 | Lung specific eQTL | Hao *et al*. single tissue lung eQTL |
| PLEKHG4B | Lung specific eQTL | Hao *et al*. single tissue lung eQTL |
| MYL4 | Lung specific eQTL | Hao *et al*. single tissue lung eQTL |
| SNAP29 | Lung specific eQTL | Hao *et al*. single tissue lung eQTL |
| WDR55 | Lung specific eQTL | Hao *et al*. single tissue lung eQTL |
| COG7 | Lung specific eQTL | Hao *et al*. single tissue lung eQTL |
| EARS2 | Lung specific eQTL | Hao *et al*. single tissue lung eQTL |
| PCDHA2 | Lung specific eQTL | Hao *et al*. single tissue lung eQTL |
| MS4A6A | Lung specific eQTL | Hao *et al*. single tissue lung eQTL |
| SLC35E4 | Lung specific eQTL | Hao *et al*. single tissue lung eQTL |
| IREB2 | Lung specific eQTL | Hao *et al*. single tissue lung eQTL |
| AVEN | Lung specific eQTL | Hao *et al*. single tissue lung eQTL |
| SKIV2L | Lung specific eQTL | Hao *et al*. single tissue lung eQTL |
| USP44 | Lung specific eQTL | Hao *et al*. single tissue lung eQTL |
| DPEP2 | Lung specific eQTL | Hao *et al*. single tissue lung eQTL |
| DUSP18 | Lung specific eQTL | Hao *et al*. single tissue lung eQTL |
| AVPR1A | Lung specific eQTL | Hao *et al*. single tissue lung eQTL |
| GGA2 | Lung specific eQTL | Hao *et al*. single tissue lung eQTL |
| PMM2 | Lung specific eQTL | Hao *et al*. single tissue lung eQTL |
| RPRML | Lung specific eQTL | Hao *et al*. single tissue lung eQTL |
| LOC648987 | Lung specific eQTL | Hao *et al*. single tissue lung eQTL |
| FGF17 | Lung specific eQTL | Hao *et al*. single tissue lung eQTL |
| IK | Lung specific eQTL | Hao *et al*. single tissue lung eQTL |
| FMN1 | Lung specific eQTL | Hao *et al*. single tissue lung eQTL |
| GPR110 | Lung specific eQTL | Hao *et al*. single tissue lung eQTL |
| SLC25A17 | Lung specific eQTL | Hao *et al*. single tissue lung eQTL |
| TSC1 | Lung specific eQTL | Hao *et al*. single tissue lung eQTL |
| NDUFA2 | Lung specific eQTL | Hao *et al*. single tissue lung eQTL |
| CCDC127 | Lung specific eQTL | Hao *et al*. single tissue lung eQTL |
| KIF1B | permissive enhancer | FANTOM5 enhancer target gene |
| ALCAM | permissive enhancer | FANTOM5 enhancer target gene |
| EXOC3 | permissive enhancer | FANTOM5 enhancer target gene |
| SLC12A7 | permissive enhancer | FANTOM5 enhancer target gene |
| LPCAT1 | permissive enhancer | FANTOM5 enhancer target gene |
| NFKBIL1 | permissive enhancer | FANTOM5 enhancer target gene |
| C6orf27 | permissive enhancer | FANTOM5 enhancer target gene |
| ATP6V1G2 | permissive enhancer | FANTOM5 enhancer target gene |
| MCCD1 | permissive enhancer | FANTOM5 enhancer target gene |
| HSPA1L | permissive enhancer | FANTOM5 enhancer target gene |
| GPANK1 | permissive enhancer | FANTOM5 enhancer target gene |
| BAG6 | permissive enhancer | FANTOM5 enhancer target gene |
| HLA-DRA | permissive enhancer | FANTOM5 enhancer target gene |
| HLA-DRB5 | permissive enhancer | FANTOM5 enhancer target gene |
| CCHCR1 | permissive enhancer | FANTOM5 enhancer target gene |
| HLA-DRB1 | permissive enhancer | FANTOM5 enhancer target gene |
| HLA-DQA1 | permissive enhancer | FANTOM5 enhancer target gene |
| HLA-DQB1 | permissive enhancer | FANTOM5 enhancer target gene |
| TAP2 | permissive enhancer | FANTOM5 enhancer target gene |
| PSMB9 | permissive enhancer | FANTOM5 enhancer target gene |
| PSMB8 | permissive enhancer | FANTOM5 enhancer target gene |
| HLA-DPB1 | permissive enhancer | FANTOM5 enhancer target gene |
| HLA-DPA1 | permissive enhancer | FANTOM5 enhancer target gene |
| HLA-DOB | permissive enhancer | FANTOM5 enhancer target gene |
| HLA-DOA | permissive enhancer | FANTOM5 enhancer target gene |
| HLA-DMB | permissive enhancer | FANTOM5 enhancer target gene |
| HLA-DMA | permissive enhancer | FANTOM5 enhancer target gene |
| BRD2 | permissive enhancer | FANTOM5 enhancer target gene |
| TAP1 | permissive enhancer | FANTOM5 enhancer target gene |
| HLA-DQB2 | permissive enhancer | FANTOM5 enhancer target gene |
| HLA-DQA2 | permissive enhancer | FANTOM5 enhancer target gene |
| PON1 | permissive enhancer | FANTOM5 enhancer target gene |
| PON3 | permissive enhancer | FANTOM5 enhancer target gene |
| PDK4 | permissive enhancer | FANTOM5 enhancer target gene |
| PHYHIP | permissive enhancer | FANTOM5 enhancer target gene |
| LGI3 | permissive enhancer | FANTOM5 enhancer target gene |
| EPB49 | permissive enhancer | FANTOM5 enhancer target gene |
| NPM2 | permissive enhancer | FANTOM5 enhancer target gene |
| TSPAN14 | permissive enhancer | FANTOM5 enhancer target gene |
| SH2D4B | permissive enhancer | FANTOM5 enhancer target gene |
| PATL1 | permissive enhancer | FANTOM5 enhancer target gene |
| STX3 | permissive enhancer | FANTOM5 enhancer target gene |
| SLC38A2 | permissive enhancer | FANTOM5 enhancer target gene |
| SCAF11 | permissive enhancer | FANTOM5 enhancer target gene |
| FAM177A1 | permissive enhancer | FANTOM5 enhancer target gene |
| RALGAPA1 | permissive enhancer | FANTOM5 enhancer target gene |
| ZFYVE1 | permissive enhancer | FANTOM5 enhancer target gene |
| PSEN1 | permissive enhancer | FANTOM5 enhancer target gene |
| NUMB | permissive enhancer | FANTOM5 enhancer target gene |
| C14orf43 | permissive enhancer | FANTOM5 enhancer target gene |
| NFKBIA | permissive enhancer | FANTOM5 enhancer target gene |
| GREM1 | permissive enhancer | FANTOM5 enhancer target gene |
| SLCO3A1 | permissive enhancer | FANTOM5 enhancer target gene |
| SCNN1G | permissive enhancer | FANTOM5 enhancer target gene |
| GGA2 | permissive enhancer | FANTOM5 enhancer target gene |
| GAS7 | permissive enhancer | FANTOM5 enhancer target gene |
| MTMR3 | permissive enhancer | FANTOM5 enhancer target gene |
| TBC1D10A | permissive enhancer | FANTOM5 enhancer target gene |
| SLC35E4 | permissive enhancer | FANTOM5 enhancer target gene |
| SF3A1 | permissive enhancer | FANTOM5 enhancer target gene |
| DUSP18 | permissive enhancer | FANTOM5 enhancer target gene |
| CCDC157 | permissive enhancer | FANTOM5 enhancer target gene |
| TOM1 | permissive enhancer | FANTOM5 enhancer target gene |
| HMOX1 | permissive enhancer | FANTOM5 enhancer target gene |
| APOL6 | permissive enhancer | FANTOM5 enhancer target gene |
| LIF | permissive enhancer | FANTOM5 enhancer target gene |
| OSM | permissive enhancer | FANTOM5 enhancer target gene |
| MB | permissive enhancer | FANTOM5 enhancer target gene |
| KIF22 | predicted enhancer target | IM-PET IMR90 |
| SOX9 | predicted enhancer target | IM-PET IMR90 |
| DIAPH1 | predicted enhancer target | IM-PET IMR90 |
| SLC38A2 | predicted enhancer target | IM-PET IMR90 |
| YPEL5 | predicted enhancer target | IM-PET IMR90 |
| EMC4 | predicted enhancer target | IM-PET IMR90 |
| ZMAT2 | predicted enhancer target | IM-PET IMR90 |
| LPCAT1 | predicted enhancer target | IM-PET IMR90 |
| SPRED1 | predicted enhancer target | IM-PET IMR90 |
| DNAL1 | predicted enhancer target | IM-PET IMR90 |
| ANXA2R | predicted enhancer target | IM-PET IMR90 |
| AHRR | predicted enhancer target | IM-PET IMR90 |
| IFT20 | predicted enhancer target | IM-PET IMR90 |
| PLEKHJ1 | predicted enhancer target | IM-PET IMR90 |
| ARID2 | predicted enhancer target | IM-PET IMR90 |
| NF2 | predicted enhancer target | IM-PET IMR90 |
| E2F3 | predicted enhancer target | IM-PET IMR90 |
| ALDH6A1 | predicted enhancer target | IM-PET IMR90 |
| COL6A3 | predicted enhancer target | IM-PET IMR90 |
| WBP1L | predicted enhancer target | IM-PET IMR90 |
| SKIV2L | predicted enhancer target | IM-PET IMR90 |
| NEU1 | predicted enhancer target | IM-PET IMR90 |
| RP11-231C14.3 | predicted enhancer target | IM-PET IMR90 |
| DAB2IP | predicted enhancer target | IM-PET IMR90 |
| JADE2 | predicted enhancer target | IM-PET IMR90 |
| PCDHGA8 | predicted enhancer target | IM-PET IMR90 |
| KSR1 | predicted enhancer target | IM-PET IMR90 |
| LBH | predicted enhancer target | IM-PET IMR90 |
| KIF26B | predicted enhancer target | IM-PET IMR90 |
| GPR75-ASB3 | predicted enhancer target | IM-PET IMR90 |
| SLC25A37 | predicted enhancer target | IM-PET IMR90 |
| IK | predicted enhancer target | IM-PET IMR90 |
| NT5C2 | predicted enhancer target | IM-PET IMR90 |
| LDB1 | predicted enhancer target | IM-PET IMR90 |
| RP11-1008C21.2 | predicted enhancer target | IM-PET IMR90 |
| ZNF410 | predicted enhancer target | IM-PET IMR90 |
| HAUS8 | predicted enhancer target | IM-PET IMR90 |
| LINC00265 | predicted enhancer target | IM-PET IMR90 |
| BAG6 | predicted enhancer target | IM-PET IMR90 |
| HARS | predicted enhancer target | IM-PET IMR90 |
| CEP78 | predicted enhancer target | IM-PET IMR90 |
| HDAC3 | predicted enhancer target | IM-PET IMR90 |
| CNNM2 | predicted enhancer target | IM-PET IMR90 |
| PDCD11 | predicted enhancer target | IM-PET IMR90 |
| THOC5 | predicted enhancer target | IM-PET IMR90 |
| ACTR1A | predicted enhancer target | IM-PET IMR90 |
| CTD-2201E18.3 | predicted enhancer target | IM-PET IMR90 |
| EXOC3 | predicted enhancer target | IM-PET IMR90 |
| GNPDA1 | predicted enhancer target | IM-PET IMR90 |
| CLPTM1L | predicted enhancer target | IM-PET IMR90 |
| TMCO6 | predicted enhancer target | IM-PET IMR90 |
| PDCD6 | predicted enhancer target | IM-PET IMR90 |
| SDHA | predicted enhancer target | IM-PET IMR90 |
| LDB2 | predicted enhancer target | IM-PET IMR90 |
| DOK2 | predicted enhancer target | IM-PET IMR90 |
| EGR3 | predicted enhancer target | IM-PET IMR90 |
| CARD8 | predicted enhancer target | IM-PET IMR90 |
| BIN3 | predicted enhancer target | IM-PET IMR90 |
| SORBS3 | predicted enhancer target | IM-PET IMR90 |
| CCAR2 | predicted enhancer target | IM-PET IMR90 |
| XPO7 | predicted enhancer target | IM-PET IMR90 |
| TCF7 | predicted enhancer target | IM-PET IMR90 |
| CHD9 | predicted enhancer target | IM-PET NHLF |
| C5 | predicted enhancer target | IM-PET NHLF |
| SGK1 | predicted enhancer target | IM-PET NHLF |
| PCDHGC5 | predicted enhancer target | IM-PET NHLF |
| PCDHAC1 | predicted enhancer target | IM-PET NHLF |
| DIAPH1 | predicted enhancer target | IM-PET NHLF |
| IDH3A | predicted enhancer target | IM-PET NHLF |
| ATP6V1G2 | predicted enhancer target | IM-PET NHLF |
| PCDHGC3 | predicted enhancer target | IM-PET NHLF |
| ANXA2R | predicted enhancer target | IM-PET NHLF |
| VARS2 | predicted enhancer target | IM-PET NHLF |
| MOB4 | predicted enhancer target | IM-PET NHLF |
| METAP2 | predicted enhancer target | IM-PET NHLF |
| UQCR10 | predicted enhancer target | IM-PET NHLF |
| ZNRF3 | predicted enhancer target | IM-PET NHLF |
| SLK | predicted enhancer target | IM-PET NHLF |
| SRA1 | predicted enhancer target | IM-PET NHLF |
| NFKB2 | predicted enhancer target | IM-PET NHLF |
| DXO | predicted enhancer target | IM-PET NHLF |
| NF2 | predicted enhancer target | IM-PET NHLF |
| CDK13 | predicted enhancer target | IM-PET NHLF |
| XBP1 | predicted enhancer target | IM-PET NHLF |
| EYA4 | predicted enhancer target | IM-PET NHLF |
| NUMB | predicted enhancer target | IM-PET NHLF |
| LDB1 | predicted enhancer target | IM-PET NHLF |
| TBPL1 | predicted enhancer target | IM-PET NHLF |
| MOXD1 | predicted enhancer target | IM-PET NHLF |
| INA | predicted enhancer target | IM-PET NHLF |
| WBP1L | predicted enhancer target | IM-PET NHLF |
| SUFU | predicted enhancer target | IM-PET NHLF |
| GBF1 | predicted enhancer target | IM-PET NHLF |
| KCTD20 | predicted enhancer target | IM-PET NHLF |
| GSN | predicted enhancer target | IM-PET NHLF |
| CNTRL | predicted enhancer target | IM-PET NHLF |
| EIF4G3 | predicted enhancer target | IM-PET NHLF |
| AGER | predicted enhancer target | IM-PET NHLF |
| RNF5 | predicted enhancer target | IM-PET NHLF |
| FKBPL | predicted enhancer target | IM-PET NHLF |
| EHMT2 | predicted enhancer target | IM-PET NHLF |
| VWA7 | predicted enhancer target | IM-PET NHLF |
| CLIC1 | predicted enhancer target | IM-PET NHLF |
| CSNK2B | predicted enhancer target | IM-PET NHLF |
| CSNK2B-LY6G5B-1181 | predicted enhancer target | IM-PET NHLF |
| GPANK1 | predicted enhancer target | IM-PET NHLF |
| BAG6 | predicted enhancer target | IM-PET NHLF |
| ATP6V1G2-DDX39B | predicted enhancer target | IM-PET NHLF |
| BTRC | predicted enhancer target | IM-PET NHLF |
| SFXN3 | predicted enhancer target | IM-PET NHLF |
| CBLB | predicted enhancer target | IM-PET NHLF |
| PPT2 | predicted enhancer target | IM-PET NHLF |
| MYO9B | predicted enhancer target | IM-PET NHLF |
| GNAQ | predicted enhancer target | IM-PET NHLF |
| MORC2 | predicted enhancer target | IM-PET NHLF |
| PGBD4 | predicted enhancer target | IM-PET NHLF |
| EWSR1 | predicted enhancer target | IM-PET NHLF |
| PCDHGB7 | predicted enhancer target | IM-PET NHLF |
| PCDHGA8 | predicted enhancer target | IM-PET NHLF |
| RHBDD3 | predicted enhancer target | IM-PET NHLF |
| TOX3 | predicted enhancer target | IM-PET NHLF |
| HSPE1 | predicted enhancer target | IM-PET NHLF |
| LBH | predicted enhancer target | IM-PET NHLF |
| HSPD1 | predicted enhancer target | IM-PET NHLF |
| PPT2-EGFL8 | predicted enhancer target | IM-PET NHLF |
| CFB | predicted enhancer target | IM-PET NHLF |
| ANO6 | predicted enhancer target | IM-PET NHLF |
| PLCL1 | predicted enhancer target | IM-PET NHLF |
| C4A | predicted enhancer target | IM-PET NHLF |
| RP11-160C18.4 | predicted enhancer target | IM-PET NHLF |
| HARS2 | predicted enhancer target | IM-PET NHLF |
| ANKHD1 | predicted enhancer target | IM-PET NHLF |
| HARS | predicted enhancer target | IM-PET NHLF |
| SF3A1 | predicted enhancer target | IM-PET NHLF |
| GAL3ST1 | predicted enhancer target | IM-PET NHLF |
| RP11-345J4.8 | predicted enhancer target | IM-PET NHLF |
| BABAM1 | predicted enhancer target | IM-PET NHLF |
| AC037459.4 | predicted enhancer target | IM-PET NHLF |
| TMEM132A | predicted enhancer target | IM-PET NHLF |
| TBC1D2B | predicted enhancer target | IM-PET NHLF |
| PRRC2A | predicted enhancer target | IM-PET NHLF |
| DDX39B | predicted enhancer target | IM-PET NHLF |
| STK19 | predicted enhancer target | IM-PET NHLF |
| HDAC3 | predicted enhancer target | IM-PET NHLF |
| ABHD16A | predicted enhancer target | IM-PET NHLF |
| PDCD11 | predicted enhancer target | IM-PET NHLF |
| LY6G5B | predicted enhancer target | IM-PET NHLF |
| RP11-127L20.5 | predicted enhancer target | IM-PET NHLF |
| FBXW2 | predicted enhancer target | IM-PET NHLF |
| ZFAND3 | predicted enhancer target | IM-PET NHLF |
| LCLAT1 | predicted enhancer target | IM-PET NHLF |
| PIM1 | predicted enhancer target | IM-PET NHLF |
| ACTR1A | predicted enhancer target | IM-PET NHLF |
| NELFE | predicted enhancer target | IM-PET NHLF |
| GTF2H4 | predicted enhancer target | IM-PET NHLF |
| CUEDC2 | predicted enhancer target | IM-PET NHLF |
| MSH5 | predicted enhancer target | IM-PET NHLF |
| COX20 | predicted enhancer target | IM-PET NHLF |
| IK | predicted enhancer target | IM-PET NHLF |
| APBB3 | predicted enhancer target | IM-PET NHLF |
| WDR55 | predicted enhancer target | IM-PET NHLF |
| CLPTM1L | predicted enhancer target | IM-PET NHLF |
| SDHA | predicted enhancer target | IM-PET NHLF |
| SRPK1 | predicted enhancer target | IM-PET NHLF |
| ZNF131 | predicted enhancer target | IM-PET NHLF |
| PDCD6 | predicted enhancer target | IM-PET NHLF |
| PCDHA4 | predicted enhancer target | IM-PET NHLF |
| AHRR | predicted enhancer target | IM-PET NHLF |
| MDGA1 | predicted enhancer target | IM-PET NHLF |
| PCDHGA6 | predicted enhancer target | IM-PET NHLF |
| FCHSD1 | predicted enhancer target | IM-PET NHLF |
| CTD-2410N18.4 | predicted enhancer target | IM-PET NHLF |
| POLR3D | predicted enhancer target | IM-PET NHLF |
| CARD8 | predicted enhancer target | IM-PET NHLF |
| PCDHGB4 | predicted enhancer target | IM-PET NHLF |
| RBM25 | predicted enhancer target | IM-PET NHLF |
| DAAM2 | predicted enhancer target | IM-PET NHLF |
| PITX3 | predicted enhancer target | IM-PET NHLF |
| NMT1 | predicted enhancer target | IM-PET NHLF |

**Table S4.** Final germline-regulated genes for SCLC.

| Gene_symbol | Regulatory_cateogry | Data_source |
| --- | --- | --- |
| VPS13A | Lung specific eQTL | GTEx single tissue V6 Lung eQTL |
| DHRS4L2 | Lung specific eQTL | GTEx single tissue V6 Lung eQTL |
| RP11-290F20.1 | Lung specific eQTL | GTEx single tissue V6 Lung eQTL |
| FAM43B | Lung specific eQTL | GTEx single tissue V6 Lung eQTL |
| SEC14L4 | Lung specific eQTL | GTEx single tissue V6 Lung eQTL |
| CTSF | Lung specific eQTL | GTEx single tissue V6 Lung eQTL |
| NMRK1 | Lung specific eQTL | GTEx single tissue V6 Lung eQTL |
| MYL4 | Lung specific eQTL | GTEx single tissue V6 Lung eQTL |
| DEPTOR | Lung specific eQTL | GTEx single tissue V6 Lung eQTL |
| TIPIN | Lung specific eQTL | GTEx single tissue V6 Lung eQTL |
| CHRNA5 | Lung specific eQTL | GTEx single tissue V6 Lung eQTL |
| HCG4P5 | Lung specific eQTL | GTEx single tissue V6 Lung eQTL |
| PDCD6IP | Lung specific eQTL | GTEx single tissue V6 Lung eQTL |
| DHRS4 | Lung specific eQTL | GTEx single tissue V6 Lung eQTL |
| RP11-109L13.1 | Lung specific eQTL | GTEx single tissue V6 Lung eQTL |
| IP6K3 | Lung specific eQTL | GTEx single tissue V6 Lung eQTL |
| TDRD6 | Lung specific eQTL | GTEx single tissue V6 Lung eQTL |
| ADHFE1 | Lung specific eQTL | GTEx single tissue V6 Lung eQTL |
| HLA-A | Lung specific eQTL | GTEx single tissue V6 Lung eQTL |
| DHRS4-AS1 | Lung specific eQTL | GTEx single tissue V6 Lung eQTL |
| RNPEP | Lung specific eQTL | GTEx single tissue V6 Lung eQTL |
| RPF2 | Lung specific eQTL | GTEx single tissue V6 Lung eQTL |
| RP11-650L12.2 | Lung specific eQTL | GTEx single tissue V6 Lung eQTL |
| ARPC5 | Lung specific eQTL | GTEx single tissue V6 Lung eQTL |
| HLA-K | Lung specific eQTL | GTEx single tissue V6 Lung eQTL |
| KAT8 | Lung specific eQTL | GTEx single tissue V6 Lung eQTL |
| JAG1 | Lung specific eQTL | GTEx single tissue V6 Lung eQTL |
| EHD2 | Lung specific eQTL | GTEx single tissue V6 Lung eQTL |
| SLC25A27 | Lung specific eQTL | GTEx single tissue V6 Lung eQTL |
| SLC44A5 | Lung specific eQTL | GTEx single tissue V6 Lung eQTL |
| KRTAP5-AS1 | Lung specific eQTL | GTEx single tissue V6 Lung eQTL |
| STX17 | Lung specific eQTL | GTEx single tissue V6 Lung eQTL |
| AC093323.3 | Lung specific eQTL | GTEx single tissue V6 Lung eQTL |
| ZFP57 | Lung specific eQTL | GTEx single tissue V6 Lung eQTL |
| RP4-539M6.14 | Lung specific eQTL | GTEx single tissue V6 Lung eQTL |
| EHD2 | Lung eQTL | GTEx multi-tissue eQTL in lung > 0.80 |
| PSMA4 | Lung eQTL | GTEx multi-tissue eQTL in lung > 0.80 |
| TIPIN | Lung eQTL | GTEx multi-tissue eQTL in lung > 0.80 |
| NLRC4 | Lung eQTL | GTEx multi-tissue eQTL in lung > 0.80 |
| ITPR3 | Lung eQTL | GTEx multi-tissue eQTL in lung > 0.80 |
| GMEB2 | Lung eQTL | GTEx multi-tissue eQTL in lung > 0.80 |
| GLTSCR2 | Lung eQTL | GTEx multi-tissue eQTL in lung > 0.80 |
| NMRK1 | Lung eQTL | GTEx multi-tissue eQTL in lung > 0.80 |
| TRIM3 | Lung eQTL | GTEx multi-tissue eQTL in lung > 0.80 |
| CCDC92 | Lung eQTL | GTEx multi-tissue eQTL in lung > 0.80 |
| INVS | Lung eQTL | GTEx multi-tissue eQTL in lung > 0.80 |
| AMD1 | Lung eQTL | GTEx multi-tissue eQTL in lung > 0.80 |
| ATG14 | Lung eQTL | GTEx multi-tissue eQTL in lung > 0.80 |
| TIMM10B | Lung eQTL | GTEx multi-tissue eQTL in lung > 0.80 |
| STX17 | Lung eQTL | GTEx multi-tissue eQTL in lung > 0.80 |
| DSCC1 | Lung eQTL | GTEx multi-tissue eQTL in lung > 0.80 |
| ZNF287 | Lung eQTL | GTEx multi-tissue eQTL in lung > 0.80 |
| MGST3 | Lung eQTL | GTEx multi-tissue eQTL in lung > 0.80 |
| PLA2G7 | Lung eQTL | GTEx multi-tissue eQTL in lung > 0.80 |
| ADHFE1 | Lung eQTL | GTEx multi-tissue eQTL in lung > 0.80 |
| SLC30A6 | Lung eQTL | GTEx multi-tissue eQTL in lung > 0.80 |
| SLC25A27 | Lung eQTL | GTEx multi-tissue eQTL in lung > 0.80 |
| CDK19 | Lung eQTL | GTEx multi-tissue eQTL in lung > 0.80 |
| DEPTOR | Lung eQTL | GTEx multi-tissue eQTL in lung > 0.80 |
| DHRS4 | Lung eQTL | GTEx multi-tissue eQTL in lung > 0.80 |
| IP6K3 | Lung eQTL | GTEx multi-tissue eQTL in lung > 0.80 |
| ARPC5 | Lung eQTL | GTEx multi-tissue eQTL in lung > 0.80 |
| S100P | Lung eQTL | GTEx multi-tissue eQTL in lung > 0.80 |
| CHRNA5 | Lung eQTL | GTEx multi-tissue eQTL in lung > 0.80 |
| PDCD6IP | Lung eQTL | GTEx multi-tissue eQTL in lung > 0.80 |
| AC093323.3 | Lung eQTL | GTEx multi-tissue eQTL in lung > 0.80 |
| OSCAR | Lung eQTL | GTEx multi-tissue eQTL in lung > 0.80 |
| LRRC34 | Lung eQTL | GTEx multi-tissue eQTL in lung > 0.80 |
| RNPEP | Lung eQTL | GTEx multi-tissue eQTL in lung > 0.80 |
| SEPW1 | Lung eQTL | GTEx multi-tissue eQTL in lung > 0.80 |
| RPRML | Lung eQTL | GTEx multi-tissue eQTL in lung > 0.80 |
| DHRS4L2 | Lung eQTL | GTEx multi-tissue eQTL in lung > 0.80 |
| DHRS4-AS1 | Lung eQTL | GTEx multi-tissue eQTL in lung > 0.80 |
| VPS13A | Lung eQTL | GTEx multi-tissue eQTL in lung > 0.80 |
| RP11-290F20.1 | Lung eQTL | GTEx multi-tissue eQTL in lung > 0.80 |
| METTL6 | Lung eQTL | GTEx multi-tissue eQTL in lung > 0.80 |
| RP11-539L10.3 | Lung eQTL | GTEx multi-tissue eQTL in lung > 0.80 |
| RP11-109L13.1 | Lung eQTL | GTEx multi-tissue eQTL in lung > 0.80 |
| DPP3 | Lung eQTL | GTEx multi-tissue eQTL in lung > 0.80 |
| RP11-887P2.6 | Lung eQTL | GTEx multi-tissue eQTL in lung > 0.80 |
| RP11-454L9.2 | Lung eQTL | GTEx multi-tissue eQTL in lung > 0.80 |
| RP11-158M2.5 | Lung eQTL | GTEx multi-tissue eQTL in lung > 0.80 |
| RP11-158M2.2 | Lung eQTL | GTEx multi-tissue eQTL in lung > 0.80 |
| RP11-158M2.4 | Lung eQTL | GTEx multi-tissue eQTL in lung > 0.80 |
| RP11-650L12.2 | Lung eQTL | GTEx multi-tissue eQTL in lung > 0.80 |
| RP11-156P1.3 | Lung eQTL | GTEx multi-tissue eQTL in lung > 0.80 |
| LRRC37A17P | Lung eQTL | GTEx multi-tissue eQTL in lung > 0.80 |
| GLTSCR2 | Lung specific eQTL | Hao single tissue lung eQTL |
| FAM91A1 | Lung specific eQTL | Hao single tissue lung eQTL |
| MYL4 | Lung specific eQTL | Hao single tissue lung eQTL |
| METTL6 | Lung specific eQTL | Hao single tissue lung eQTL |
| DST | Lung specific eQTL | Hao single tissue lung eQTL |
| TNN | Lung specific eQTL | Hao single tissue lung eQTL |
| CDK19 | Lung specific eQTL | Hao single tissue lung eQTL |
| RPRML | Lung specific eQTL | Hao single tissue lung eQTL |
| KCNK6 | Lung specific eQTL | Hao single tissue lung eQTL |
| ARPM1 | Lung specific eQTL | Hao single tissue lung eQTL |
| WLS | Lung specific eQTL | Hao single tissue lung eQTL |
| FXC1 | Lung specific eQTL | Hao single tissue lung eQTL |
| SYF2 | permissive enhancer | FANTOM5 enhancer target gene |
| C1orf63 | permissive enhancer | FANTOM5 enhancer target gene |
| ELF3 | permissive enhancer | FANTOM5 enhancer target gene |
| LGR6 | permissive enhancer | FANTOM5 enhancer target gene |
| EPRS | permissive enhancer | FANTOM5 enhancer target gene |
| BPNT1 | permissive enhancer | FANTOM5 enhancer target gene |
| RUNX3 | permissive enhancer | FANTOM5 enhancer target gene |
| RAB3GAP2 | permissive enhancer | FANTOM5 enhancer target gene |
| ZAK | permissive enhancer | FANTOM5 enhancer target gene |
| SLC7A11 | permissive enhancer | FANTOM5 enhancer target gene |
| ZDHHC21 | permissive enhancer | FANTOM5 enhancer target gene |
| NFIB | permissive enhancer | FANTOM5 enhancer target gene |
| C9orf72 | permissive enhancer | FANTOM5 enhancer target gene |
| CYP26A1 | permissive enhancer | FANTOM5 enhancer target gene |
| SIK3 | permissive enhancer | FANTOM5 enhancer target gene |
| PCSK7 | permissive enhancer | FANTOM5 enhancer target gene |
| C12orf44 | permissive enhancer | FANTOM5 enhancer target gene |
| KRT80 | permissive enhancer | FANTOM5 enhancer target gene |
| KRT7 | permissive enhancer | FANTOM5 enhancer target gene |
| RCBTB2 | permissive enhancer | FANTOM5 enhancer target gene |
| FNDC3A | permissive enhancer | FANTOM5 enhancer target gene |
| AKAP13 | permissive enhancer | FANTOM5 enhancer target gene |
| C19orf33 | permissive enhancer | FANTOM5 enhancer target gene |
| PSMD8 | permissive enhancer | FANTOM5 enhancer target gene |
| PAK4 | permissive enhancer | FANTOM5 enhancer target gene |
| MRPS12 | permissive enhancer | FANTOM5 enhancer target gene |
| FBXO27 | permissive enhancer | FANTOM5 enhancer target gene |
| ACTN4 | permissive enhancer | FANTOM5 enhancer target gene |
| APP | permissive enhancer | FANTOM5 enhancer target gene |
| MGAT3 | permissive enhancer | FANTOM5 enhancer target gene |
| PXMP4 | predicted enhancer target | IM-PET IMR90 |
| LIF | predicted enhancer target | IM-PET IMR90 |
| ACTN4 | predicted enhancer target | IM-PET IMR90 |
| NAPA | predicted enhancer target | IM-PET IMR90 |
| SLC44A3 | predicted enhancer target | IM-PET IMR90 |
| ADHFE1 | predicted enhancer target | IM-PET IMR90 |
| RRS1 | predicted enhancer target | IM-PET IMR90 |
| MTMR3 | predicted enhancer target | IM-PET IMR90 |
| SNRPEP3 | predicted enhancer target | IM-PET IMR90 |
| NF2 | predicted enhancer target | IM-PET IMR90 |
| SLC25A18 | predicted enhancer target | IM-PET IMR90 |
| BAHD1 | predicted enhancer target | IM-PET IMR90 |
| RUFY3 | predicted enhancer target | IM-PET IMR90 |
| WDHD1 | predicted enhancer target | IM-PET IMR90 |
| RP4-639F20.1 | predicted enhancer target | IM-PET IMR90 |
| ARFIP2 | predicted enhancer target | IM-PET IMR90 |
| LINC01057 | predicted enhancer target | IM-PET IMR90 |
| NIN | predicted enhancer target | IM-PET IMR90 |
| RP11-290F20.1 | predicted enhancer target | IM-PET IMR90 |
| KRT80 | predicted enhancer target | IM-PET IMR90 |
| TNIK | predicted enhancer target | IM-PET IMR90 |
| ATP6V1E1 | predicted enhancer target | IM-PET IMR90 |
| SIPA1L3 | predicted enhancer target | IM-PET IMR90 |
| EDEM1 | predicted enhancer target | IM-PET IMR90 |
| RP11-539L10.2 | predicted enhancer target | IM-PET IMR90 |
| SEPW1 | predicted enhancer target | IM-PET IMR90 |
| LEMD2 | predicted enhancer target | IM-PET IMR90 |
| TMEM155 | predicted enhancer target | IM-PET IMR90 |
| SIDT2 | predicted enhancer target | IM-PET IMR90 |
| PCSK7 | predicted enhancer target | IM-PET IMR90 |
| RNF214 | predicted enhancer target | IM-PET IMR90 |
| TIMM10B | predicted enhancer target | IM-PET IMR90 |
| EIF3K | predicted enhancer target | IM-PET IMR90 |
| CNIH1 | predicted enhancer target | IM-PET NHLF |
| CGRRF1 | predicted enhancer target | IM-PET NHLF |
| RUFY3 | predicted enhancer target | IM-PET NHLF |
| PCDHGA12 | predicted enhancer target | IM-PET NHLF |
| ACTN4 | predicted enhancer target | IM-PET NHLF |
| ARFIP2 | predicted enhancer target | IM-PET NHLF |
| MECOM | predicted enhancer target | IM-PET NHLF |
| CRH | predicted enhancer target | IM-PET NHLF |
| ADHFE1 | predicted enhancer target | IM-PET NHLF |
| ZNF215 | predicted enhancer target | IM-PET NHLF |
| SIDT2 | predicted enhancer target | IM-PET NHLF |
| OXSM | predicted enhancer target | IM-PET NHLF |
| S100P | predicted enhancer target | IM-PET NHLF |
| IDH3A | predicted enhancer target | IM-PET NHLF |
| RNF214 | predicted enhancer target | IM-PET NHLF |
| SPINT2 | predicted enhancer target | IM-PET NHLF |
| C8orf46 | predicted enhancer target | IM-PET NHLF |
| SPAST | predicted enhancer target | IM-PET NHLF |
| PAK4 | predicted enhancer target | IM-PET NHLF |
| BPNT1 | predicted enhancer target | IM-PET NHLF |
| SLC25A18 | predicted enhancer target | IM-PET NHLF |
| ACTRT3 | predicted enhancer target | IM-PET NHLF |
| DNHD1 | predicted enhancer target | IM-PET NHLF |
| MYNN | predicted enhancer target | IM-PET NHLF |
| STRN3 | predicted enhancer target | IM-PET NHLF |
| SAMD4A | predicted enhancer target | IM-PET NHLF |
| WDHD1 | predicted enhancer target | IM-PET NHLF |
| RGL1 | predicted enhancer target | IM-PET NHLF |
| CNN3 | predicted enhancer target | IM-PET NHLF |
| DPM1 | predicted enhancer target | IM-PET NHLF |
| SPRY1 | predicted enhancer target | IM-PET NHLF |
| PCDHGA11 | predicted enhancer target | IM-PET NHLF |
| FER1L6 | predicted enhancer target | IM-PET NHLF |
| SIRT2 | predicted enhancer target | IM-PET NHLF |
| MLTK | predicted enhancer target | IM-PET NHLF |
| REV3L | predicted enhancer target | IM-PET NHLF |
| ZHX3 | predicted enhancer target | IM-PET NHLF |
| DCAF17 | predicted enhancer target | IM-PET NHLF |
| KIAA1109 | predicted enhancer target | IM-PET NHLF |
| DST | predicted enhancer target | IM-PET NHLF |
| GNPDA1 | predicted enhancer target | IM-PET NHLF |
| TBC1D2B | predicted enhancer target | IM-PET NHLF |
| INVS | predicted enhancer target | IM-PET NHLF |
| DPF1 | predicted enhancer target | IM-PET NHLF |
| UBE2V1 | predicted enhancer target | IM-PET NHLF |
| NECAB3 | predicted enhancer target | IM-PET NHLF |
| TMCO1 | predicted enhancer target | IM-PET NHLF |
| NIN | predicted enhancer target | IM-PET NHLF |
| SIK3 | predicted enhancer target | IM-PET NHLF |
| GTPBP1 | predicted enhancer target | IM-PET NHLF |
| RNF14 | predicted enhancer target | IM-PET NHLF |
| FAM91A1 | predicted enhancer target | IM-PET NHLF |
| MTSS1 | predicted enhancer target | IM-PET NHLF |
| MYBL1 | predicted enhancer target | IM-PET NHLF |
| ILK | predicted enhancer target | IM-PET NHLF |
| PCSK7 | predicted enhancer target | IM-PET NHLF |
| CEP164 | predicted enhancer target | IM-PET NHLF |
| RRP8 | predicted enhancer target | IM-PET NHLF |
| CDKN3 | predicted enhancer target | IM-PET NHLF |

**Table S5.** Final set of germline-regulated genes from GWAS Catalog SNPs.

| Gene symbol | Regulatory category | Data source |
| --- | --- | --- |
| *CHRNA5* | Lung specific eQTL | GTEx single tissue V6 Lung eQTL |
| *RP11-650L12.2* | Lung specific eQTL | GTEx single tissue V6 Lung eQTL |
| *TP63* | Lung specific eQTL | GTEx single tissue V6 Lung eQTL |
| *PSMA4* | lung tissue eQTL | GTEx multi-tissue eQTL in lung > 0.80 |
| *CHRNA5* | lung tissue eQTL | GTEx multi-tissue eQTL in lung > 0.80 |
| *RP11-650L12.2* | lung tissue eQTL | GTEx multi-tissue eQTL in lung > 0.80 |
| *ZSCAN29* | lung tissue eQTL | GTEx multi-tissue eQTL in lung > 0.80 |
| *TP63* | Lung specific eQTL | Hao *et al*. single tissue lung eQTL |
| *CLPTM1L* | predicted enhancer target | IM-PET IMR90 |
| *CLPTM1L* | predicted enhancer target | IM-PET NHLF |

**Table S6.** Pathway enrichment results for LUAD.

| **KEGG**  **pathway name** | **# enriched genes**  **in pathway** | **Gene symbol** | **Unique genomic**  **regions** | **# genes**  **in pathway** | **Raw p** | **BH Adjusted p** |
| --- | --- | --- | --- | --- | --- | --- |
| Metabolic pathways | 17 | *HIBADH, NT5C2, PIGN, GAPDH, AMT, IDH3A, IMPDH2, MDH2, ACOX1, QARS, POLR3D, UGCG, UGT2B4, LPCAT1, COX4I2, GGPS1, CD38* | 15 | 1130 | 1.49E-05 | 0.0006 |
| Tight junction | 4 | *MYH9, MYH4, CLDN23, LLGL2* | 4 | 132 | 0.003 | 0.024 |
| Viral myocarditis | 3 | *MYH9, MYH4, CAV1* | 3 | 70 | 0.0039 | 0.024 |
| Endocytosis | 5 | *NEDD4L, CAV1, ASAP1, CAV2, CHMP6* | 4 | 201 | 0.0022 | 0.024 |
| Bacterial invasion  of epithelial cells | 3 | *CAV1, SHC4, CAV2* | 2 | 70 | 0.0039 | 0.024 |
| Insulin signaling pathway | 4 | *TSC1, SHC4, FOXO1, PRKAR1A* | 4 | 138 | 0.0035 | 0.024 |
| Nicotinate and  nicotinamide metabolism | 2 | *NT5C2, CD38* | 2 | 24 | 0.0052 | 0.0275 |
| Apoptosis | 3 | *ENDOD1, BCL2L1, PRKAR1A* | 3 | 87 | 0.0071 | 0.0328 |
| Citrate cycle (TCA cycle) | 2 | *IDH3A, MDH2* | 2 | 30 | 0.008 | 0.0329 |
| Focal adhesion | 4 | *LAMB2, CAV1, SHC4, CAV2* | 3 | 200 | 0.0127 | 0.047 |
| Proteasome | 2 | *PSMA4, PSMD14* | 2 | 44 | 0.0167 | 0.0492 |
| Lysosome | 3 | *GGA3, GGA1, CTSH* | 3 | 121 | 0.0173 | 0.0492 |
| Aldosterone-regulated  sodium reabsorption | 2 | *NEDD4L, SFN* | 2 | 42 | 0.0153 | 0.0492 |

**Table S7.** Pathway enrichment results for LUSC.

| **KEGG pathway name** | **# enriched genes**  **in pathway** | **Gene symbol** | **Unique genomic**  **regions** | **# genes in**  **pathway** | **Raw p** | **BH Adjusted p** |
| --- | --- | --- | --- | --- | --- | --- |
| Staphylococcus aureus infection | 15 | *HLA-DRB1, HLA-DRA, HLA-DMA, HLA-DPA1, HLA-DQA2, HLA-DQA1, HLA-DQB1, C4A, HLA-DMB, C5, CFB, HLA-DOA, HLA-DOB, HLA-DPB1, HLA-DRB5* | 2 | 55 | 1.44E-20 | 1.15E-18 |
| Asthma | 12 | *HLA-DRB1, HLA-DRA, HLA-DMA, HLA-DPA1, HLA-DQA2, HLA-DQA1, HLA-DQB1, HLA-DMB, HLA-DOA, HLA-DOB, HLA-DRB5, HLA-DPB1* | 1 | 30 | 4.65E-19 | 1.86E-17 |
| Type I diabetes mellitus | 13 | *HLA-DRB1, HLA-DRA, HLA-DMA, HLA-DPA1, HLA-DQA2, HLA-DQA1, HLA-DQB1, HLA-DMB, HLA-DOA, HSPD1, HLA-DOB, HLA-DRB5, HLA-DPB1* | 2 | 43 | 1.17E-18 | 3.12E-17 |
| Antigen processing and presentation | 15 | *HLA-DRB1, HLA-DRA, HSPA1L, HLA-DMA, HLA-DPA1, HLA-DQA2, HLA-DQA1, HLA-DQB1, HLA-DMB, TAP1, TAP2, HLA-DOA, HLA-DOB, HLA-DPB1, HLA-DRB5* | 1 | 76 | 3.04E-18 | 6.08E-17 |
| Allograft rejection | 12 | *HLA-DRB1, HLA-DRA, HLA-DMA, HLA-DPA1, HLA-DQA2, HLA-DQA1, HLA-DQB1, HLA-DMB, HLA-DOA, HLA-DOB, HLA-DRB5, HLA-DPB1* | 1 | 37 | 9.56E-18 | 1.53E-16 |
| Graft-versus-host disease | 12 | *HLA-DRB1, HLA-DRA, HLA-DMA, HLA-DPA1, HLA-DQA2, HLA-DQA1, HLA-DQB1, HLA-DMB, HLA-DOA, HLA-DOB, HLA-DRB5, HLA-DPB1* | 1 | 41 | 3.98E-17 | 5.31E-16 |
| Intestinal immune network for IgA production | 12 | *HLA-DRB1, HLA-DRA, HLA-DMA, HLA-DPA1, HLA-DQA2, HLA-DQA1, HLA-DQB1, HLA-DMB, HLA-DOA, HLA-DOB, HLA-DRB5, HLA-DPB1* | 1 | 48 | 3.37E-16 | 3.85E-15 |
| Autoimmune thyroid disease | 12 | *HLA-DRB1, HLA-DRA, HLA-DMA, HLA-DPA1, HLA-DQA2, HLA-DQA1, HLA-DQB1, HLA-DMB, HLA-DOA, HLA-DOB, HLA-DRB5, HLA-DPB1* | 1 | 52 | 9.75E-16 | 9.75E-15 |
| Viral myocarditis | 13 | *HLA-DRB1, HLA-DRA, HLA-DMA, HLA-DPA1, HLA-DQA2, HLA-DQA1, HLA-DQB1, HLA-DMB, EIF4G3, HLA-DOA, HLA-DOB, HLA-DRB5, HLA-DPB1* | 2 | 70 | 1.29E-15 | 1.15E-14 |
| Leishmaniasis | 13 | *HLA-DRB1, HLA-DRA, HLA-DMA, HLA-DPA1, HLA-DQA2, HLA-DQA1, HLA-DQB1, NFKBIA, HLA-DMB, HLA-DOA, HLA-DOB, HLA-DRB5, HLA-DPB1* | 2 | 72 | 1.91E-15 | 1.53E-14 |
| Rheumatoid arthritis | 13 | *HLA-DRB1, HLA-DRA, HLA-DMA, HLA-DPA1, HLA-DQA2, HLA-DQA1, HLA-DQB1, ATP6V1G2, HLA-DMB, HLA-DOA, HLA-DOB, HLA-DRB5, HLA-DPB1* | 1 | 91 | 4.61E-14 | 3.35E-13 |
| Phagosome | 15 | *HLA-DRB1, HLA-DRA, HLA-DMA, HLA-DPA1, HLA-DQA2, HLA-DQA1, HLA-DQB1, ATP6V1G2, HLA-DMB, TAP1, TAP2, HLA-DOA, HLA-DOB, HLA-DPB1, HLA-DRB5* | 1 | 153 | 1.50E-13 | 1.00E-12 |
| Toxoplasmosis | 14 | *HLA-DRB1, HLA-DRA, HSPA1L, HLA-DMA, HLA-DPA1, HLA-DQA2, HLA-DQA1, HLA-DQB1, NFKBIA, HLA-DMB, HLA-DOA, HLA-DOB, HLA-DRB5, HLA-DPB1* | 2 | 132 | 3.30E-13 | 2.03E-12 |
| Systemic lupus erythematosus | 14 | *HLA-DRB1, HLA-DRA, HLA-DMA, HLA-DPA1, HLA-DQA2, HLA-DQA1, HLA-DQB1, C4A, HLA-DMB, C5, HLA-DOA, HLA-DOB, HLA-DPB1, HLA-DRB5* | 2 | 136 | 5.00E-13 | 2.86E-12 |
| Cell adhesion molecules (CAMs) | 13 | *HLA-DRB1, HLA-DRA, HLA-DMA, HLA-DPA1, HLA-DQA2, HLA-DQA1, HLA-DQB1, HLA-DMB, HLA-DOA, ALCAM, HLA-DOB, HLA-DRB5, HLA-DPB1* | 2 | 133 | 6.70E-12 | 3.57E-11 |
| Spliceosome | 6 | *HSPA1L, PPIL1, RBM25, ZMAT2, SF3A1, DDX39B* | 5 | 127 | 0.0002 | 0.0009 |
| Metabolic pathways | 19 | *PPT2, EARS2, ALDH6A1, GNPDA1, IDH3A, UQCR10, PMM2, GAL3ST1, LCLAT1, PON1, SDHA, NT5C2, PON2, POLR3D, NDUFA2, PON3, ATP6V1G2, LPCAT1, CYP17A1* | 14 | 1130 | 0.0002 | 0.0009 |
| Wnt signaling pathway | 6 | *TCF7, CSNK2B, NFATC3, PSEN1, BTRC, DAAM2* | 6 | 150 | 0.0005 | 0.0022 |
| Aminoacyl-tRNA biosynthesis | 4 | *HARS2, VARS2, EARS2, HARS* | 3 | 63 | 0.0008 | 0.0034 |
| Huntington's disease | 6 | *GNAQ, DNAL1, NDUFA2, SDHA, UQCR10, TBPL1* | 6 | 183 | 0.0015 | 0.006 |
| Proteasome | 3 | *PSMB8, PSMB9, PSMA4* | 2 | 44 | 0.0032 | 0.0122 |
| Jak-STAT signaling pathway | 5 | *LIF, SPRED1, PIM1, CBLB, OSM* | 4 | 155 | 0.0039 | 0.0142 |
| Alzheimer's disease | 5 | *GNAQ, NDUFA2, PSEN1, SDHA, UQCR10* | 5 | 167 | 0.0054 | 0.0188 |
| Pathways in cancer | 7 | *TCF7, FGF17, SUFU, NFKB2, NFKBIA, E2F3, CBLB* | 6 | 326 | 0.0065 | 0.0217 |
| NOD-like receptor signaling pathway | 3 | *CARD8, NFKBIA, NLRC4* | 3 | 58 | 0.0069 | 0.0221 |
| Shigellosis | 3 | *DIAPH1, BTRC, NFKBIA* | 3 | 61 | 0.0079 | 0.0243 |
| Biosynthesis of unsaturated fatty acids | 2 | *ACOT2, ACOT1* | 1 | 21 | 0.0085 | 0.0252 |
| RNA degradation | 3 | *SKIV2L, HSPD1, PATL1* | 3 | 71 | 0.0119 | 0.0317 |
| Complement and coagulation cascades | 3 | *C5, CFB, C4A* | 2 | 69 | 0.0111 | 0.0317 |
| Oxidative phosphorylation | 4 | *NDUFA2, ATP6V1G2, SDHA, UQCR10* | 4 | 132 | 0.0119 | 0.0317 |
| Chronic myeloid leukemia | 3 | *NFKBIA, E2F3, CBLB* | 3 | 73 | 0.0129 | 0.0333 |
| Collecting duct acid secretion | 2 | *SLC12A7, ATP6V1G2* | 2 | 27 | 0.0139 | 0.0347 |
| Citrate cycle (TCA cycle) | 2 | *IDH3A, SDHA* | 2 | 30 | 0.017 | 0.0412 |
| Hematopoietic cell lineage | 3 | *HLA-DRB1, HLA-DRA, HLA-DRB5* | 1 | 88 | 0.0211 | 0.0496 |
| Prostate cancer | 3 | *TCF7, NFKBIA, E2F3* | 3 | 89 | 0.0218 | 0.0498 |

**Table S8.** Pathway enrichment results for SCLC.

| **KEGG**  **pathway name** | **# enriched genes**  **in pathway** | **Gene symbol** | **Unique genomic regions** | **# genes**  **in pathway** | **Raw p** | **BH adjusted p** |
| --- | --- | --- | --- | --- | --- | --- |
| Metabolic pathways | 13 | *MGAT3, EPRS, REV3L,*  *DHRS4L2, PLA2G7, DPM1, GNPDA1, IDH3A, AMD1, OXSM, BPNT1, ATP6V1E1, DHRS4* | 10 | 1130 | 0.0002 | 0.003 |
| Retinol metabolism | 3 | *DHRS4L2, DHRS4, CYP26A1* | 2 | 64 | 0.0015 | 0.0112 |
| Focal adhesion | 4 | *ACTN4, TNN, ILK, PAK4* | 3 | 200 | 0.0055 | 0.0275 |
| Proteasome | 2 | *PSMD8, PSMA4* | 2 | 44 | 0.0105 | 0.0387 |
| N-Glycan biosynthesis | 2 | *MGAT3, DPM1* | 2 | 49 | 0.0129 | 0.0387 |

**Table S9.** Independent locus level analysis for LUAD unique.

| Regulatory category | # unique genes | # chromosomes | # total unique regions |
| --- | --- | --- | --- |
| GTEx single tissue V6 Lung eQTL | 41 | 14 | 25 |
| GTEx multi-tissue eQTL in lung > 0.80 | 43 | 18 | 28 |
| GTEx combined | 66 | 18 | 37 |
| Hao single tissue lung eQTL | 23 | 12 | 18 |
| FANTOM5 enhancer target gene | 31 | 12 | 15 |
| IM-PET IMR90 | 43 | 12 | 20 |
| IM-PET NHLF | 80 | 15 | 28 |
| IM-PET combined | 105 | 16 | 34 |
| All genes | 193 | 21 | 69 |

**Table S10.** Independent locus level analysis for LUSC unique.

| Regulatory category | # unique genes | # chromosomes | # total unique regions |
| --- | --- | --- | --- |
| GTEx single tissue V6 Lung eQTL | 47 | 14 | 23 |
| GTEx multi-tissue eQTL in lung > 0.80 | 72 | 18 | 36 |
| GTEx combined | 95 | 18 | 42 |
| Hao single tissue lung eQTL | 26 | 9 | 17 |
| FANTOM5 enhancer target gene | 63 | 13 | 16 |
| IM-PET IMR90 | 59 | 16 | 27 |
| IM-PET NHLF | 110 | 16 | 28 |
| IM-PET combined | 150 | 18 | 37 |
| All genes | 287 | 19 | 71 |

**Table S11.** Independent locus level analysis for SCLC unique.

| Regulatory category | # unique genes | # chromosomes | # total unique regions |
| --- | --- | --- | --- |
| GTEx single tissue V6 Lung eQTL | 29 | 13 | 25 |
| GTEx multi-tissue eQTL in lung > 0.80 | 45 | 14 | 30 |
| GTEx combined | 56 | 16 | 37 |
| Hao single tissue lung eQTL | 10 | 6 | 10 |
| FANTOM5 enhancer target gene | 30 | 12 | 15 |
| IM-PET IMR90 | 32 | 13 | 21 |
| IM-PET NHLF | 56 | 13 | 29 |
| IM-PET combined | 77 | 16 | 38 |
| All genes | 154 | 20 | 69 |

**Table S12.** Independent locus level analysis for LUAD overlap LUSC.

| Regulatory category | # unique genes | # chromosomes | # total unique regions |
| --- | --- | --- | --- |
| GTEx single tissue V6 Lung eQTL | 0 | 0 | 0 |
| GTEx multi-tissue eQTL in lung > 0.80 | 2 | 2 | 2 |
| GTEx combined | 2 | 2 | 2 |
| Hao single tissue lung eQTL | 1 | 1 | 1 |
| FANTOM5 enhancer target gene | 5 | 2 | 2 |
| IM-PET IMR90 | 2 | 2 | 2 |
| IM-PET NHLF | 7 | 3 | 3 |
| IM-PET combined | 7 | 3 | 3 |
| All genes | 16 | 6 | 6 |

**Table S13.** Independent locus level analysis for LUAD overlap SCLC.

| Regulatory category | # unique genes | # chromosomes | # total unique regions |
| --- | --- | --- | --- |
| GTEx single tissue V6 Lung eQTL | 0 | 0 | 0 |
| GTEx multi-tissue eQTL in lung > 0.80 | 0 | 0 | 0 |
| GTEx combined | 0 | 0 | 0 |
| Hao single tissue lung eQTL | 0 | 0 | 0 |
| FANTOM5 enhancer target gene | 0 | 0 | 0 |
| IM-PET IMR90 | 0 | 0 | 0 |
| IM-PET NHLF | 1 | 1 | 1 |
| IM-PET combined | 1 | 1 | 1 |
| All genes | 1 | 1 | 1 |

**Table S14.** Independent locus level analysis for LUSC overlap SCLC.

| Regulatory category | # unique genes | # chromosomes | # total unique regions |
| --- | --- | --- | --- |
| GTEx single tissue V6 Lung eQTL | 4 | 2 | 2 |
| GTEx multi-tissue eQTL in lung > 0.80 | 4 | 2 | 2 |
| GTEx combined | 8 | 3 | 3 |
| Hao single tissue lung eQTL | 2 | 1 | 1 |
| FANTOM5 enhancer target gene | 0 | 0 | 0 |
| IM-PET IMR90 | 1 | 1 | 1 |
| IM-PET NHLF | 2 | 1 | 1 |
| IM-PET combined | 2 | 2 | 2 |
| All genes | 12 | 5 | 5 |

**Table S15.** Independent locus level analysis for all overlap.

| Regulatory category | # unique genes | # chromosomes | # total unique regions |
| --- | --- | --- | --- |
| GTEx single tissue V6 Lung eQTL | 2 | 1 | 1 |
| GTEx multi-tissue eQTL in lung > 0.80 | 3 | 1 | 1 |
| GTEx combined | 3 | 1 | 1 |
| Hao single tissue lung eQTL | 0 | 0 | 0 |
| FANTOM5 enhancer target gene | 0 | 0 | 0 |
| IM-PET IMR90 | 0 | 0 | 0 |
| IM-PET NHLF | 2 | 1 | 1 |
| IM-PET combined | 2 | 1 | 1 |
| All genes | 5 | 1 | 1 |


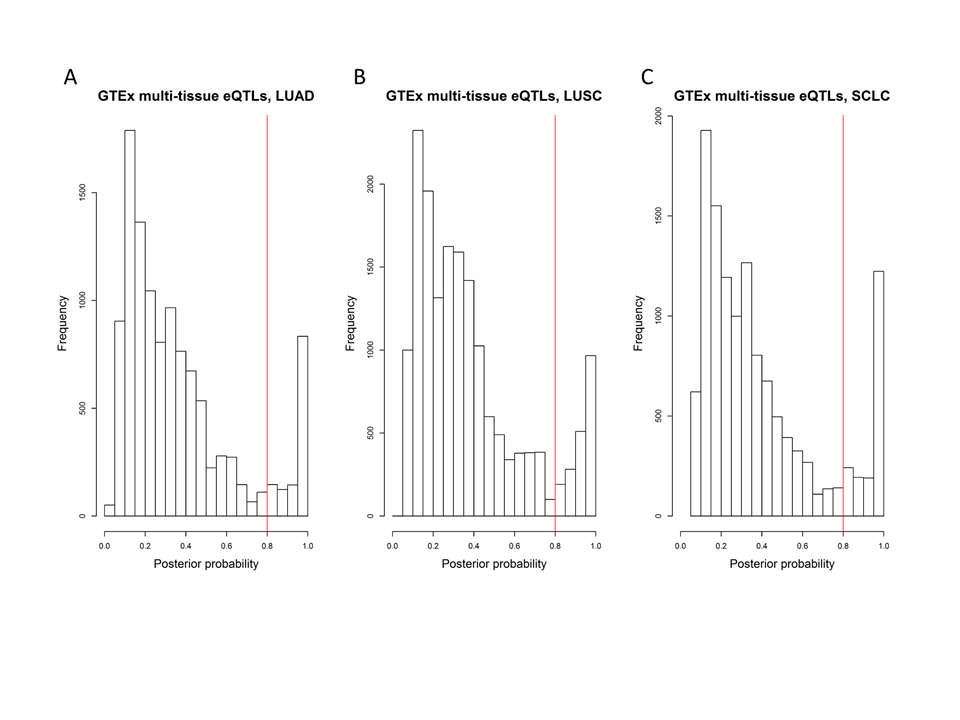


**Figure S1. Determination of significance for GTEx multi-tissue eQTLs.** Posterior probabilities in lung tissue for all multi-tissue eQTLs are plotted for each subtype. The posterior probabilities of the eQTLs for each subtype, LUAD (A), LUSC (B) and SCLC (C), resemble a bimodal distribution. We chose a significance threshold to capture the second distribution of values. Red line indicates the cutoff used of a posterior probability of 0.8.


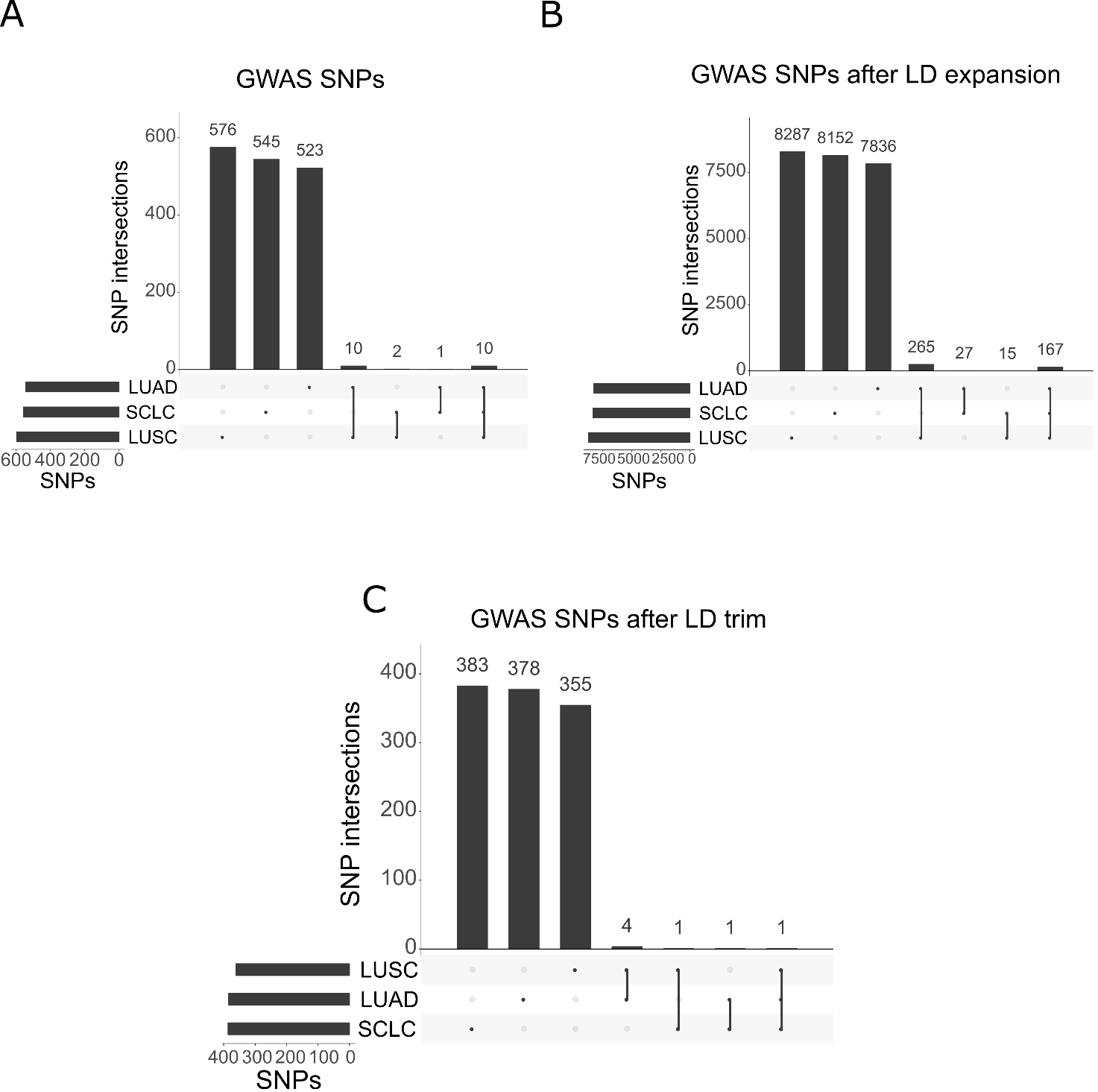


**Figure S2. Comparison of SNPs from the GWAS for lung cancer.** UpSetR plots show overlap of SNPs found in the GWAS (p < 10^-3^) for lung cancer by subtype in (A), after LD (r^2^ > 0.8 within 1Mb) expansion in (B), and after LD trim in (C). Overall, few SNPs are shared between all three subtypes.


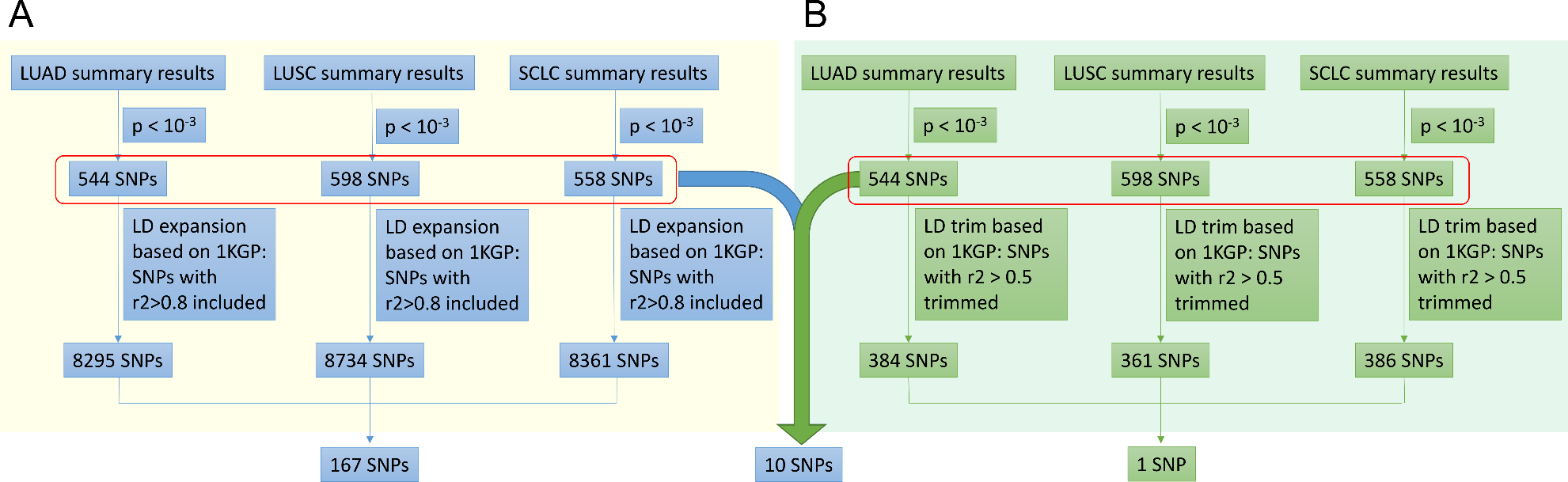


**Figure S3.** **Pipelines used to obtain overlap in the LD expanded and LD trimmed SNPs per lung cancer subtype.** Panel A in yellow shows the SNPs used for LD expansion. These SNPs were also used to obtain the overlap of 167 SNPs. Panel B in green shows the SNPs used for LD trim. These SNPs were used to obtain the overlap of one SNP. The arrow indicates the 10 overlapping SNPs from the original GWAS results used in for both LD expansion and LD trim. 1KGP: 1000 Genomes Project.


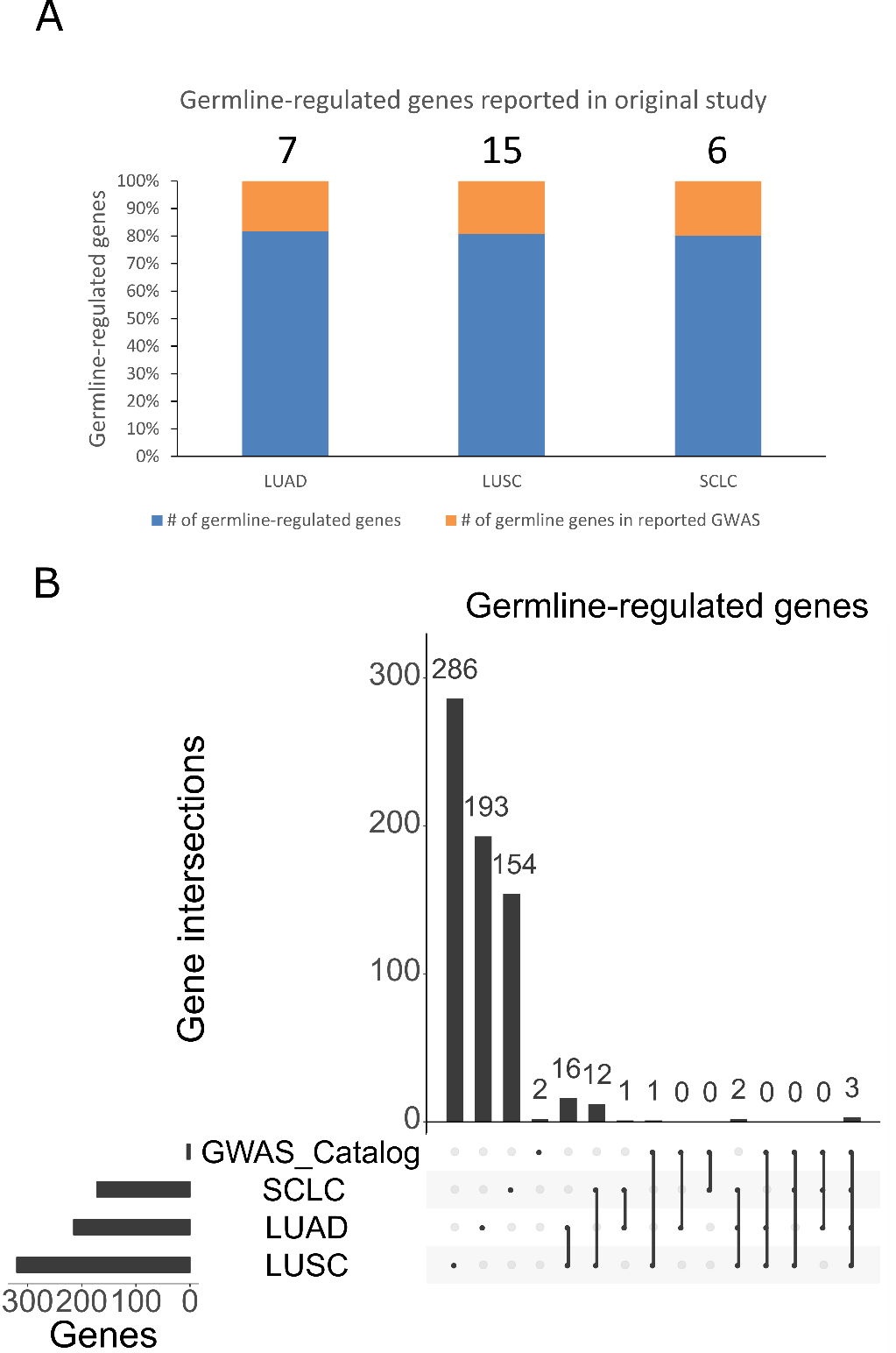


**Figure S4. Comparison of germline-regulated genes to original report and the GWAS Catalog.** In panel A, we show the number and percentage of the germline-regulated genes we discovered using the functional genomics pipeline with the SNPs that were reported in the initial GWAS at (p < 1 × 10^-4^). The numbers above the bars are the total number of germline-regulated genes found in this study that were originally reported. The majority of germline-regulated genes discovered in this study were initially missed due to reporting based only upon physical location. Panel B shows the overlap of germline-regulated genes found using SNPs from the GWAS Catalog with the three lung cancer subtypes.


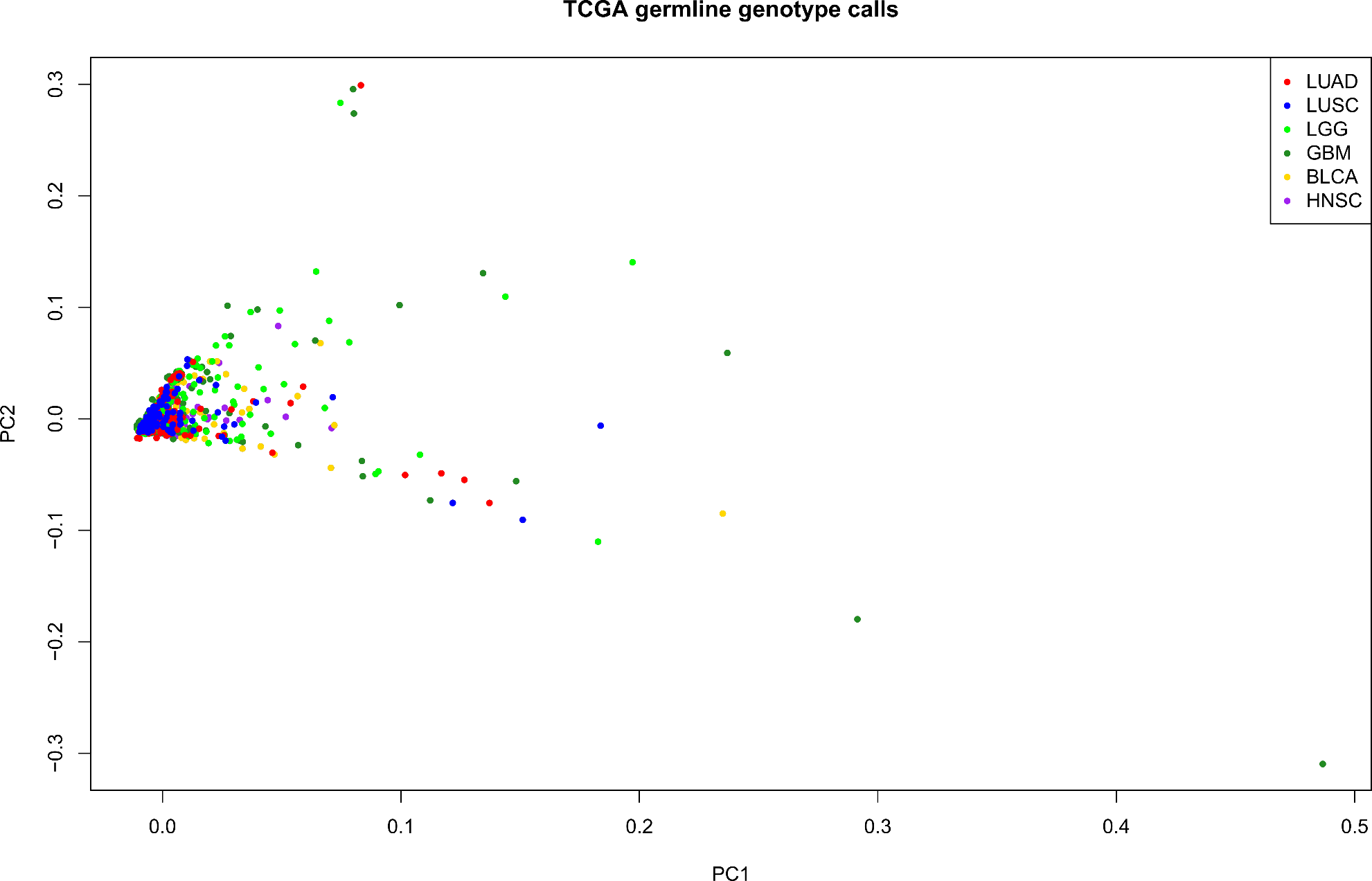


**Figure S5. PCA of germline genotype data from TCGA in six cancer types.** The top two principal components are plotted based upon the eigenvectors calculated from PLINK. LUAD is plotted in red and LUSC is plotted in blue.
